# Confidence through consensus: a neural mechanism for uncertainty monitoring

**DOI:** 10.1038/srep21830

**Published:** 2016-02-24

**Authors:** Luciano Paz, Andrea Insabato, Ariel Zylberberg, Gustavo Deco, Mariano Sigman

**Affiliations:** 1 Integrative Neuroscience Laboratory, IFIBA, CONICET and Physics Department, FCEyN, UBA, Buenos Aires, Argentina; 2 Universidad Pompeu Fabra, Barcelona, Spain; 3 Department of Neuroscience, Howard Hughes Medical Institute, Columbia University, New York, NY 10032, USA; 4 Universidad Torcuato di Tella, Buenos Aires, Argentina

## Abstract

Models that integrate sensory evidence to a threshold can explain task accuracy, response times and confidence, yet it is still unclear how confidence is encoded in the brain. Classic models assume that confidence is encoded in some form of balance between the evidence integrated in favor and against the selected option. However, recent experiments that measure the sensory evidence’s influence on choice and confidence contradict these classic models. We propose that the decision is taken by many loosely coupled modules each of which represent a stochastic sample of the sensory evidence integral. Confidence is then encoded in the dispersion between modules. We show that our proposal can account for the well established relations between confidence, and stimuli discriminability and reaction times, as well as the fluctuations influence on choice and confidence.

The decision-making process has been widely modeled as a stochastic integration of sensory evidence to a threshold^1^,^2^,^3^,^4^,^5^. These models have been used to explain with quantitative detail task accuracy, response times and confidence^6^,^7^,^8^,^9^, yet it is still unclear how confidence is encoded in the brain. A classic model by Vickers^10^ assumes that, when deciding amongst alternatives based on sensory evidence, the evidence in favor of each option is noisily integrated until one reaches a threshold. After this race, the option that reached the threshold first is selected and the balance of evidence between competing alternatives encodes the confidence. On the other hand, a typical model that is derived from optimal statistical decision theory, encodes a single decision variable that integrates the difference between the evidences in favor of each alternative^11^,^12^. This variable is related to the log odds of each alternative being correct given the evidence so far and follows a drift-diffusion process until a threshold of admissibility. The confidence is then given by the belief of having made a correct response.

Both models have been used to successfully explain several behavioral aspects of decision-making confidence, the most noteworthy being the relations between confidence and task difficulty, and confidence and response times^8^,^13^,^14^,^15^. Typically, confidence is low for difficult trials and high for easy trials, and if subjects are free to respond whenever they choose, their confidence is higher for fast responses^16^,^17^,^18^,^19^,^20^,^21^,^22^,^23^. Both models also have some shortcomings, for example they are not well defined to study confidence in more than two alternative tasks. Furthermore, recent psychophysical experiments have been able to measure the influence that the sensory evidence has on the resulting decision and confidence as a function of time^24^. By letting external noise rather than internal noise limit task performance, the average of the noise conditioned by the subject’s choice provides an estimate of the integration window and the sensitivity of decisions to fluctuations as a function of time (i.e. the influence that sensory evidence at a given time has on choice)^25^. This average is referred to as the decision and confidence kernels. The main characteristics of these kernels (at least for two alternatives tasks) are that evidence fluctuations in favor of an option, have the same influence on the resulting decision as fluctuations against the competing alternative. This is not the case for confidence, where fluctuations in favor of an option yield an increase in high confidence report rates, whereas fluctuations against the alternative option almost do not affect confidence. This property is in staggering contradiction with what drift-diffusion and balance of evidence models of confidence predict^24^. Drift-diffusion is inherently symmetric in the sense that fluctuations in favor of an option immediately become fluctuations against the competing option. The balance of evidence also fails as it takes into account the difference between accumulated evidence for each option to construct confidence reports, thus being symmetrically affected by fluctuations.

In this work we propose a model that is able to explain both the decision and confidence kernels. We make a key assumption based on the fact that the integration of evidence is corrupted by internal and external noise^26^. This implies that the integral that drives choice will be randomly distributed. If subjects have access to a representation of the former distribution (such as a summary statistic, like the distribution’s mean and variance), the belief that the decision is correct can be estimated. This belief will depend on the reliability of the evidence, and we assume it is normatively associated to decision confidence through the dispersion of the distribution of evidence integrals^27^. For instance, a highly reliable stimulus will be normatively associated to higher confidence reports whereas low reliability will produce low confidence reports. Furthermore, we ground our proposal on three key relations.

1. In stochastic processes, the variance of the evidence’s integral is very correlated with elapsed time^28^ (See SI section A). Hence, by relying on the dispersion to construct confidence reports, confidence will be very correlated to response times.
2. Attractor neural networks have been shown to implement evidence integration for probabilistic decision-making^29^,^30^. These attractors rely on the competition of two populations that encode the competing options and receive sensory input in favor of their encoded alternative. Competition is mediated by the dynamics of slow recurrent excitatory feedback, to integrate fast sensory input, and lateral inhibition, to force an option’s selection. Fluctuations of the sensory evidence have an asymmetric effect on the attractor’s response time, as a consequence of two mechanisms: 1) positive fluctuations bring more total input current to the network compared to negative fluctuations and 2) the sensory input to one population affects its firing rate instantly but it takes more time to affect the dynamics of the competing population.
3. There is evidence that supports the coexistence of multiple integration processes in the brain, even for the same sensory cue^31^,^32^,^33^. For example, Lafuente *et al.*^33^ show in a random dot movement task that MIP and LIP integrate the evidence when the decision is to reach to a location, whilst LIP also integrates when the decision is signaled by saccades. We also propose that multiple almost independent integration processes coexist in the same brain region.

We propose that the decision process is performed redundantly and in parallel by many loosely coupled modules that integrate the time varying sensory input. These modules form a sample of the of evidence integral’s probability distribution and hence can be used to estimate its variance, which in turn is used to report confidence. Furthermore, we propose that each module is an ANN. We hypothesize that the fluctuations’ asymmetric influence on each ANN’s time should transfer to an asymmetric influence on the dispersion of samples, leading to asymmetric confidence kernels.

Our proposal is similar to Koriat’s self-consistency model^34^ in which subjects are assumed to retrieve a sample of representations of the alternatives, decide based on a majority rule, and assess their confidence based on the consistency of samples. Koriat assumes that the consistency of the samples is a measure of the selected alternative’s reliability just as we assume that the dispersion is a measure of sensory reliability. However, Koriat assumes that consistency is measured from the difference between the number of samples that are in favor and against the selected option, while we use the dispersion within the samples of the selected alternative. Our model also provides a neurophysiological implementation while Koriat’s is purely normative.

Our proposal is independent of a particular implementation of the decision modules. However, we provide an exemplary computational implementation in order to address the following issues:

1. What is the maximum degree of interconnection between modules so that they can no longer be considered independent samples of the evidence integral probability distribution? To address this, we study the correlation of the inter-module dispersion, increasing an inter-module coupling parameter (*IC*).
2. How can the decision be read from the activity of the modules? We propose a “voting scheme” where each module “votes” for an option and when an option gets more than half the votes, it is selected. We provide a simple firing rate threshold detection neural implementation for detecting each module’s vote.
3. How can the variance be readout from the distribution of firing rates? We show that an indirect measure of the dispersion can be computed by counting the number of modules that have their firing rate in certain range above the vote threshold. This is equivalent to computing percentiles over the distribution of firing rates.
4. How is the variance transformed to a binary confidence report? We propose that this occurs in a separate network, which selects high or low confidence with a sigmoid probability that depends on the dispersion’s estimate.

Figure 1 shows a schematic representation of our model’s operation for decision making and confidence reports.

## Results

### Model construction

**Figure 1:**
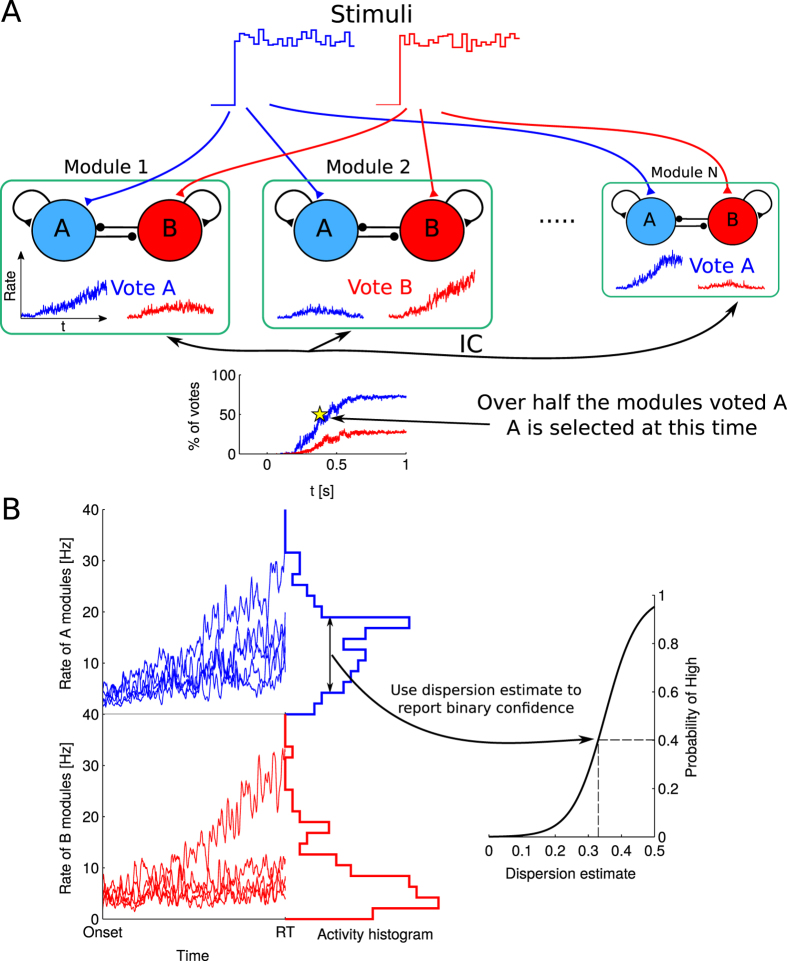
A schematic representation of the model. (**A**) shows the decision mechanism. Sensory input of the competing alternatives is fed into the *N* separate modules. Each module is constructed by an ANN with two populations that have recurrent excitation and lateral inhibition, and receive the external sensory input from one of the two alternatives, and a baseline of noisy background input from other regions of the brain that are not task specific. All the populations are interconnected across modules. This interconnection is regulated by parameter *IC*. Each module casts a vote in favor of one option depending on the competition’s outcome. When an option is voted by more than half the modules (represented with a star), it is selected as the response. (**B**) shows the confidence mechanism. All modules integrate evidence and commit to a global choice at a certain time. At that time, the dispersion amongst the selected options firing rate, *σ*_*dv*_ is estimated, and its value is transmitted to an external layer. This layer assigns the binary confidence report randomly with a sigmoid probability that depends on the dispersion estimate.

We assume that in order to decide, sensory evidence is integrated to a threshold. We assume that confidence is determined by an estimate of the sensory evidence’s reliability, which can be decoded from the dispersion of the evidence integral’s probability distribution. In order to estimate said variability we propose a network of many modules that integrate the same sensory evidence in parallel and decide collectively (Fig. 1A). We propose that each module can be thought of as a sample from the evidence integral probability distribution, thus the sensory reliability, and in turn confidence, can be decoded from an estimate of the inter-module firing rate dispersion (*σ*_*dv*_) of the populations that are associated to the selected option at response time (Fig. 1B).

Additionally, we propose that each module is an attractor neural network (ANN), a widely studied neural implementation of evidence accumulation that relies on reverberant activity of competing neural populations and mutual inhibition mediated by slow NMDA channel opening dynamics^35^,^36^,^37^,^38^. The decision of each ANN depends on the activity of the competing decision populations. When a threshold of activity (*λ*) is surpassed, the ANN commits to a choice. We study the simplified case where *λ* remains constant (we take it to be 15 Hz), although there is evidence the decision threshold varies^39^,^40^,^41^,^42^. When a module’s ANN chooses an option, it casts a vote in favor of it. The global decision is taken when an option is voted by over half the modules (Fig. 1A).

We assume that confidence is decoded from an estimate of *σ*_*dv*_ (we provide the details of the proposed estimate in sec. “A neural mechanism to decide and estimate *σ*_*dv*_”) in a separate layer. Our aim is to model binary confidence reports (i.e. high or low confidence), hence the layer that decodes confidence must produce binary values. We propose that the probability of a high confidence report is given by a sigmoid distribution that takes the *σ*_*dv*_’s estimate as input, 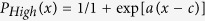. The parameters of the sigmoid control the bias and the slope of the transition from high to low confidence (Fig. 1B).

### Effect of module interconnectivity

**Figure 2:**
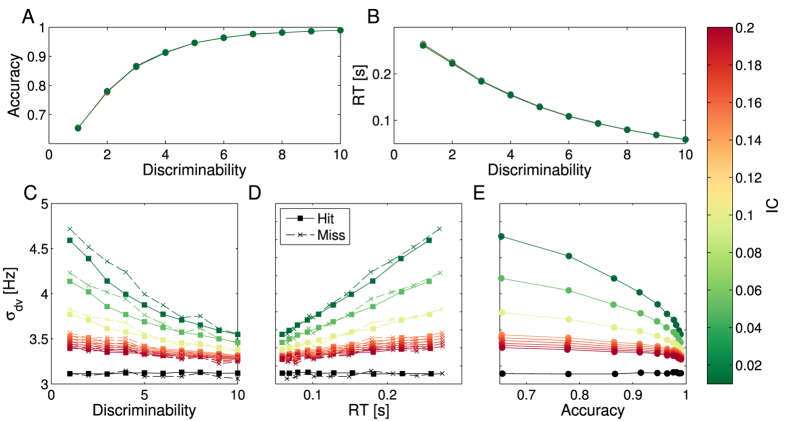
How *IC* affects *σ*_*dv*_’s ability to encode confidence. (**A**,**B**) show the model’s accuracy (i.e. the average correct trials) and response times as a function of stimuli discriminability for several *IC* values. The different *IC* values are represented with different colors as shown in the lateral color bar. The black data points are for 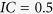. It is clear that different *IC* values do not affect the relation between these variables. (**C**–**E**) show the average *σ*_*dv*_ for different IC values as a function of discriminability, average RT and average accuracy. In (**C**,**D**), square markers indicate averaged values over correct trials and crosses correspond to averaged values over incorrect trials. It is clear that *σ*_*dv*_ is correlated with discriminability, RT and accuracy for low *IC* values, and the correlation vanishes for high *IC*’s. It is also clear that for low discriminabilities, *σ*_*dv*_ is on average higher, hence confidence will be lower. In C, it is clear that error trials have higher average *σ*_*dv*_ for all discriminabilities, and thus will be associated with lower confidence reports. However, *σ*_*dv*_’s increase in error trials does not affect the functional relation between *σ*_*dv*_ and RT, as is clear from (**D**).

In order to test our proposal, we first study how module interconnectivity (controlled by parameter *IC*) affects our assumption that *σ*_*dv*_ can be used to decode confidence. To do this, we study *σ*_*dv*_’s correlation with stimuli discriminability, task accuracy and reaction times (RT) in free to respond perceptual decisions, with varying *IC* values (Fig. 2). When *IC* = 0, modules are fully independent, while for 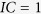 modules are fully coupled. We simulate a network of 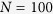 modules that must decide which of two stimuli is brighter. Both stimuli brightness flicker around a fixed mean each 40 ms. The distractor’s average luminance was fixed and the target luminance was varied. The stimuli discriminability measures the difference between the target and the distractor’s brightness. We simulated 2000 trials for each discriminability and *IC* value.

A first key result is that *IC* does not affect the mean accuracy nor the mean RT over trials as a function of stimuli discriminability (Fig. 2A,B). However, for small IC values, average *σ*_*dv*_ is strongly correlated with discriminability, task difficulty and accuracy (Fig. 2C–E). This correlation disappears for growing *IC* values. It is clear that for small *IC* values, average *σ*_*dv*_ decreases with discriminability, and increases with mean RT (in fact, the positive correlation between *σ*_*dv*_ and RT also occurs within the same discriminability level as shown in SI Fig. S6). As we assume that *σ*_*dv*_ is an inverse measure of the reliability of the stimuli, high *σ*_*dv*_ will confer on average low confidence. This implies that our model reproduces the well known positive correlation between discriminability and confidence^16^,^19^, and RT and confidence^17^,^18^. Furthermore, our model also reproduces the known positive resolution of confidence - higher confidence for correct rather than incorrect responses (Fig. 2C). However, the studied task only yields slow errors (Fig. 2D) and is not suited to study our model’s ability to predict confidence in tasks characterized by fast errors^43^,^44^.

Hence, even when the modules are interconnected, the known correlations between confidence and other relevant behavioral observables (accuracy, RT and discriminability) are preserved. However, this interconnection must be small.

### A neural mechanism to decide and estimate *σ*_*dv*_

In the previous sections we stated that the model commits to a choice based on the “votes” of all modules and decodes confidence from *σ*_*dv*_. In this section we provide a neural implementation of the decision method and *σ*_*dv*_’s estimation.

We propose that when the activity of one of the competing populations, e.g. populations sensitive to option A, in a module surpasses *λ*, the module “votes” for A. Detecting if a population has an activity greater than a certain threshold is easily accomplished using a disinhibition microcircuit^45^,^46^,^47^. Briefly, the crossing is signaled by a separate binary population of neurons that are either silent or bursting. This binary population is normally inhibited by a group of interneurons. When one of the competing populations surpasses *λ* it inhibits the interneurons and releases the inhibition of the binary population, which begins to burst. Counting votes is simply accomplished by summing the activity of the binary populations.

This decision mechanism implies that, at the response time, the median of the distribution of firing rates for the selected option is *λ*. Hence, *σ*_*dv*_ can be estimated from the from the fraction of modules in the vicinity of *λ*^48^. In particular, it is sufficient to count the fraction of modules that are between *λ* and a slightly higher value 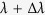 (FMC). We take *λ* = 15 Hz and Δ*λ* = 5 Hz. This is accomplished by subtracting the sum of the bursting neurons that indicate activity greater than *λ* with a second group of binary neurons that signal activity greater than 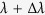 (Fig. 1B and SI Fig. S3).

**Figure 3:**
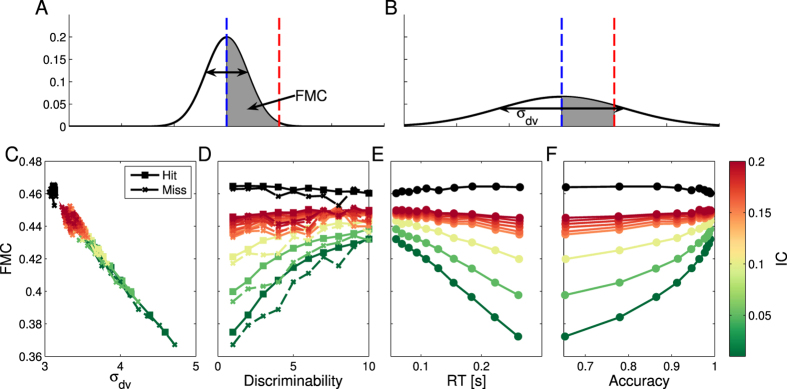
(**A**,**B**) Schematic of the relation between the fraction of modules in the counter range, FMC, and *σ*_*dv*_. The counter range is placed between *λ* and 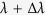. When an option is selected, the median firing rate is equal to *λ* and the FMC is inversely related to *σ*_*dv*_. (**C**) measures the relation between average *σ*_*dv*_ and average FMC for different discriminabilities and several *IC* values. The different *IC* values are represented with different colors as shown in the lateral color bar. The black data points are for 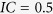. (**D**–**F**) show FMC as a function of stimuli discriminability, average RT and accuracy. It is clear that average FMC is inversely correlated to *σ*_*dv*_, hence for small *IC*’s FMC is correlated with discriminability, RT and accuracy. However, the inverse relation with *σ*_*dv*_ associates large FMC to high confidence and small FMC to low confidence.

The intuition of the mechanism is simple: if the variance is low, the firing rate of all modules should be within a narrow range relative to the median. Instead, if the variance is high, only a few modules will have firing rates within a narrow interval relative to the median (Fig. 3A,B). This relation can be empirically tested (Fig. 3C). It is clear that FMC is inversely correlated with *σ*_*dv*_ and the correlations with discriminability, RT and accuracy are preserved with an inverted dependence (Fig. 3D–F, the inverse relation exists even within the same discriminability level SI Fig. S7). Hence, FMC is a valid representation of stimuli reliability and can be used to decode confidence.

### Fluctuations’ asymmetric influence on ANNs

**Figure 4:**
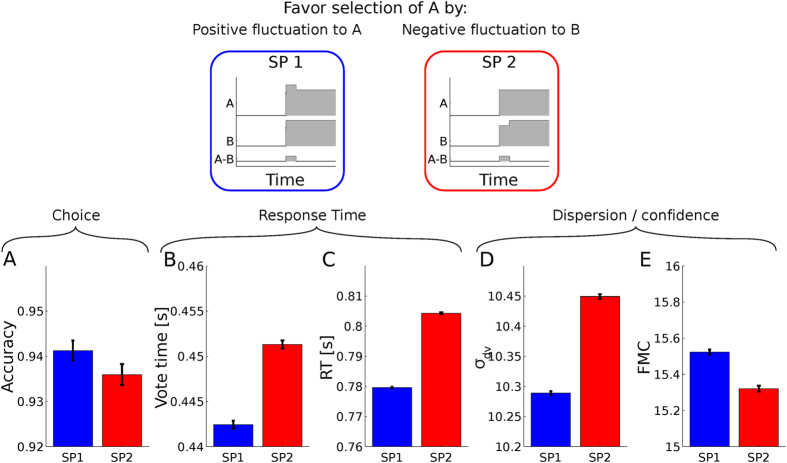
Asymmetric influence of sensory fluctuations to the model’s decision output. The top panels schematically show the two stimulation protocols (SP 1 and SP 2). SP 1 has a brief positive fluctuation in favor of (**A**), SP 2 has the a fluctuation of opposite sign and equal strength against (**B**). (**A**) Shows the network’s accuracy for both SPs. (**B**) shows a single module’s mean vote time under the both SPs. (**C**–**E**) show the network’s average RT, *σ*_*dv*_ and FMC under both SPs.

A key relation, that had not been studied previously, and which we rely on is the asymmetric influence that fluctuations have on an ANN’s RT. We hypothesize that this asymmetric influence should transfer to the whole network’s RT and to confidence, due to the correlation between *σ*_*dv*_ and RT. To illustrate this property, we simulate a network with 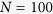 modules and measure the network RT, *σ*_*dv*_, FMC, accuracy and time at which one of the modules casts its vote (vote time) under two stimulation protocols (SP). One where a brief positive pulse (an increase of 1cd/m^2^ for 40 ms) is injected into the A population, and another SP where a pulse of the same amplitude but opposite sign is delivered to the B population (Fig. 4, the basal luminance is 50 cd/m^2^). A simulation of 10^5^ trials of each condition revealed that, as expected, both pulse manipulations resulted in comparable effects on accuracy (Fig. 4A), increasing the probability of selecting option A relative to chance for both SPs. However, the two pulse conditions had markedly different effects on RTs. For the single module’s vote time, correct votes were fastest for SP 1 (Fig. 4B).

This shows that at the module level, vote times are asymmetrically affected by sensory fluctuations. This asymmetry can be interpreted to be a consequence of the fact that a positive fluctuation brings more total input current (the combined input to both competing populations) while a negative fluctuation brings less total input. When a module has a higher total input current, it commits to a vote faster, which is in fact the basis for the neural implementation of the balance of speed and accuracy in decision making^40^,^42^. Furthermore, the asymmetric influence can also be interpreted to derive from the different latencies with which fluctuations in favor of an option become synaptic input against the competing alternative. Sensory evidence in favor of the encoded option propagates rapidly (in V1, it has been shown to be mediated by AMPA receptors^49^) and hence is integrated rapidly, while lateral interactions have a slower build-up time governed by the temporal constants of NMDA receptors and the characteristic time of recursion in the circuit^35^,^50^. Hence, this could be interpreted to cause an asymmetric influence of the fluctuations on each of the competing populations, which in turn transfers to module vote time.

We hypothesized that the fluctuations’ asymmetric influence on vote time transfers to an asymmetric influence on the network RT and dispersion. We are able to confirm our hypothesis finding that network’s RT are significantly faster for SP 1 (Fig. 4C) and *σ*_*dv*_ is significantly lower (Fig. 4D). FMC is also asymmetrically affected (Fig. 4E) reflecting smaller dispersion for SP 1, thus leading to on average higher confidence reports for SP 1 relative to SP 2. This implies that positive fluctuations that target the selected option produce on average higher confidence than negative fluctuations that target the non-selected option.

### The model accounts for subjects’ experimental performance and confidence

In this section we show that our model is able to reproduce subjects’ performance and confidence reports, and the decision and confidence kernels in a two alternative, reaction time, perceptual task.

**Figure 5:**
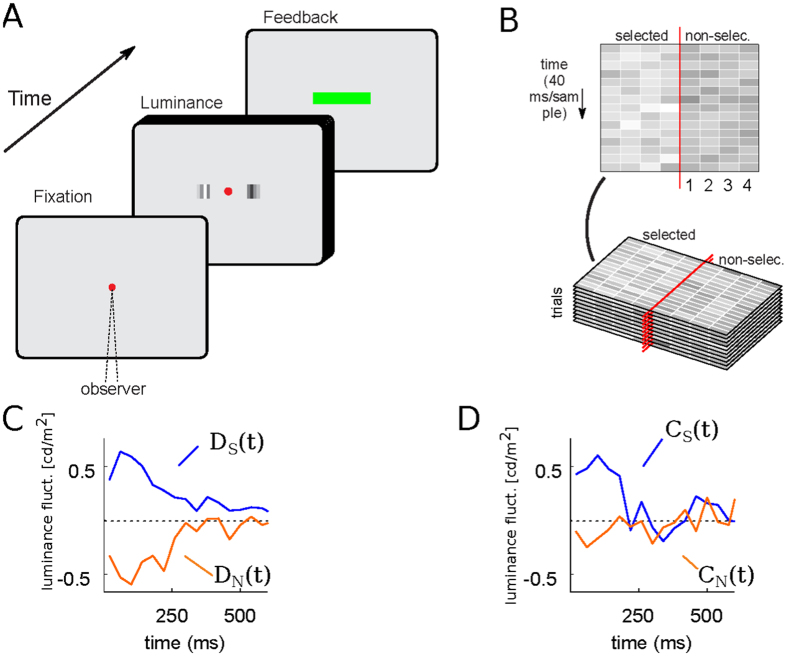
(**A**) A trial of the luminance task. Two patches of flickering bars (updated at 25 Hz) were presented until participants made a response. Participants indicated which patch is brighter and the confidence in their decision with a single manual response. (**B**) Spatiotemporal profile of the luminance signal. The red vertical line represents the fixation point, and the four columns to each side indicate the luminance in time of the four bars in each patch, numbered from the fovea to the periphery. (**C**,**D**) show the subject’s decision and confidence kernels respectively.

We aim to model the experimental data obtained in^24^. In this experiment, human subjects had to select the brightest of two patches, reporting simultaneously the choice (‘left’ or ‘right’) and the confidence in their choice (‘high’ or ‘low’) (Fig. 5A). The critical manipulation of the experiment was the addition of time-varying luminance noise to the average luminance of each patch. By letting external noise rather than internal noise limit task performance, the average of the noise conditioned by the subject’s choice provides an estimate of the integration window and the influence that sensory evidence at a given time has on choice Ahumada1996 (Fig. 5B show a sample trial’s fluctuations for the selected and non-selected patches). The average of the fluctuations only conditioned by choice gives the decision kernel (*D*_*S*_ and *D*_*N*_), and measures the influence of the fluctuation of the selected and non-selected patches on choice (Fig. 5C). By discriminating confidence reports, the confidence kernel is computed (*C*_*S*_ and *C*_*N*_), the influence of the fluctuation of the selected and non-selected patches on confidence (Fig. 5D). Zylberberg *et al.*, found a symmetrical decision kernel and an asymmetrical confidence kernel, which is inconsistent with both balance of evidence and diffusion decision confidence models^24^.

**Figure 6:**
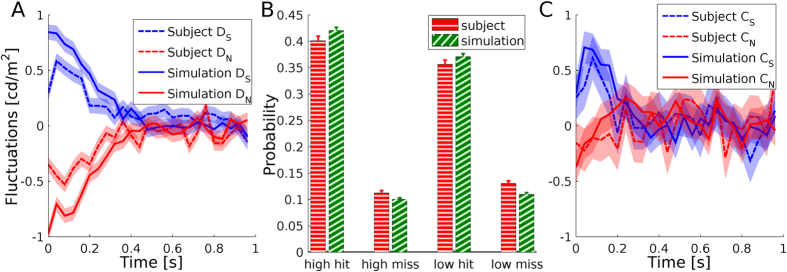
Behavioral fit results. (**A**) Subjects and model’s fitted decision kernels. (**B**) Subjects and simulations fitted accuracy grouped by confidence reports. (**C**) Subjects and model’s confidence kernels. In (**A**,**C**), time is measured from stimulus onset, and the shaded areas indicate standard deviation around the mean.

In order to show that our model of confidence yields asymmetrical confidence kernels, we fit the subjects decision kernel and task performance. To do this, we simulate a network of 100 modules that receive sensory input from two sources with the same distribution target and distractor luminances as seen by the subjects. We propose that the luminance signal is linearly transformed to neural input current as, 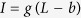, where *I* is the neural input, *L* is the observed luminance, *g* is the input gain and *b* is the input bias. We determine the values of *g* and *b* by fitting the model’s decision kernel and task performance to the subjects (Fig. 6A). After obtaining the luminance transformation parameters, we fit the parameters of the sigmoid that is used to transform FMC into a binary confidence reports. To do this, we fit the subjects performance discriminated by confidence (i.e. high-hits, high-misses, low-hits and low-misses, Fig. 6B). Crucially, the confidence kernels are not used for fitting, and thus the model’s confidence kernels can be considered a prediction. We found that the model’s confidence kernels are asymmetric and in excellent agreement with the subjects’ data (Fig. 6C).

## Discussion

In this work we presented a neural model for two alternative decisions where an ensemble of modules collectively decides and, more importantly, encodes the decision’s confidence in the dispersion of firing rates. We showed that our model is able to account for well established relations between confidence and task difficulty (higher confidence for easier trials^16^,^17^,^18^,^19^,^20^), and confidence and RT (higher confidence for faster decisions^10^,^13^,^18^,^51^). More importantly, it is able to account for the sensory evidence’s asymmetric influence on confidence^24^.

Our model’s key assumption is that decision confidence is decoded from the inter-module distribution of firing rates of the neurons encoding the selected option. We assume that the activity of each module can be interpreted as a sample of the sensory evidence’s integral over time. If subjects have access to a representation of the probability density of the sensory evidence integrals, they can estimate their belief of having made a correct decision, i.e. their confidence. This interpretation is based on the bayesian interpretation of confidence^27^. Briefly, the bayesian interpretation assumes that subjects decide following bayesian inference to infer the probability distribution (posterior) that each alternative is correct given the evidence (distributional confidence). The subjects then report a summary confidence rating that combines the information of the posterior distribution (can be either a binary - high/low - or continuous rating). In order to do this, subjects can rely on summary statistics (such as the mean and variance) instead of the entire distributional information. Our model only has access to a sample of evidence integrals, not the probability distribution, and decodes confidence from the inter-module dispersion of neurons sensitive to the selected option (a single summary statistic). We reason that it only requires this because the dispersion is strongly related to the sensory reliability, i.e. the signal to noise ratio, which by itself is a possible confidence heuristic^52^. It could however rely on more information of the samples (other statistics) to decode confidence. For instance, most models of confidence only rely on the difference between the firing rates of the selected and non-selected alternatives^53^ (similar to Vickers balance of evidence^10^). Furthermore, this difference can be directly related to the log odds measure of confidence, used by diffusion models^11^,^12^, if one assumes that neurons use a probabilistic population code^54^,^55^. The main difference with our model is that we use many modules to decide and in turn have access to many samples of the sensory evidence’s integral. Hence our model has access to more summary statistics to report confidence, and not only on the difference between competing options.

There are other proposals that also assume that confidence is determined from multiple samples, e.g Koriat’s self-consistency model^34^. However, Koriat’s consistency rule is similar to the “balance of evidence” as it constructs confidence from the difference between the number of samples that are in favor and against the selected option. Our model uses a statistic that only takes in to account the samples of the selected option, and disregards the samples of the non-selected option. Hence, our proposal, in a way, implements a confirmation bias^56^,^57^, an ubiquitous stereotypical error in human confidence judgments, where only the evidence consistent with the decision is used to report confidence.

Our main contribution is that our model is able to account for the asymmetric confidence kernels, while “balance of evidence” models cannot^24^. This is possible thanks to the asymmetric influence that sensory fluctuations have on each module’s vote time, which transfers to both network RT and inter-module dispersion. This asymmetry can be interpreted to be a consequence of the fact that a positive fluctuation brings more total input current (the combined input to both competing populations) while a negative fluctuation brings less total input. When a module has a higher total input current, it commits to a vote faster, which is in fact the basis for the neural implementation of the balance of speed and accuracy in decision making^40^,^42^. Furthermore, the asymmetric influence can also be interpreted to derive from the different latencies with which fluctuations in favor of an option become synaptic input against the competing alternative. Sensory evidence in favor of the encoded option propagates rapidly (in V1, it has been shown to be mediated by AMPA receptors^49^) and hence is integrated rapidly, while lateral interactions have a slower build-up time governed by the temporal constants of NMDA receptors and the characteristic time of recursion in the circuit^35^,^50^. Hence, this could be interpreted to cause an asymmetric influence of the fluctuations on each of the competing populations, which in turn transfers to module vote time.

Furthermore, our model is also suited to explain confidence reports in different experimental paradigms such as the fixed delay paradigm^11^,^13^,^58^. In this paradigm, subjects are presented with the stimulus during a fixed interval and are forced to decide after the stimulus is turned off. The main result is that the longer the stimulus is presented, the higher the confidence, a property that our model reproduces (SI sec. E). However, we do not study many known properties of confidence. For example, we did not study tasks characterized by fast errors^43^,^44^. These are normally linked to tasks where subjects are forced to balance their speed and accuracy tradeoff with additional costs for the passage of time. Our model’s speed-accuracy tradeoff (i.e. decision policy) can be tuned by changing the background input that targets all the competing populations (SI sec. E). However, our model is constructed assuming a constant decision policy, and thus we do not study the problem of confidence calibration^27^. Calibration is the process through which a summary statistic (in our case the *σ*_*dv*_ or FMC) is transformed to certainty level or a confidence report (which we assume occurs in a separate layer). This problem requires feedback connections and parameter tuning to actively learn the proper calibration for a variable decision policy. This also implies that the parameters that determine the probability of high confidence should also change with background input. This interesting problem is well beyond what we studied in the present work.

One of the main questions that should be addressed by future studies is: “how does the modularity arise?” Our model assumes that the network that decides is formed by modules that are by themselves networks with many neurons. It is crucial to study how this modular architecture could emerge, either from a property of the topology, sparse connectivity or synaptic plasticity. However, one of our important findings is that the modules do not need to be fully independent. Some degree of interconnection does not make the dispersion amongst module less informative, hence neurons residing in a given module can be connected to neurons outside it and the network could still function. A detailed study of the extension of our model to a spiking neuron network is necessary.

We also report a novel prediction of the model in order to falsify (or confirm) it with new experiments. The network modules are ANNs, that when stimulated, increase their firing rate until they are in the vicinity of a stable steady state that has one population with increased firing rate and the other with low firing rate. This implies that as time goes on, more modules will reach a stable steady state, and variability will decrease or reach a constant level. Our prediction is that intermodule variability should increase as a function of RT and task difficulty. This prediction should be tested in a neurophysiological experiment, with simultaneous neural recordings, where RT and confidence are observed. Furthermore, our model is built upon the idea that the decision is taken redundantly by many modules. This implies that the covariance between pairs of “integrating” neurons is not merely described by two point-processes with the same underlying rate, to some degree they must integrate evidence independently and, thus, may arrive at opposite conclusions.

## Methods

### Network model

Here we detail the network model without the neural layers that monitor choice and FMC (the full network that contains the neural populations that monitor choice and FMC is described in SI sec. B). The network contains *N* decision modules, where each module is composed of two decision populations (A and B) described by rate equations similar to^36^:


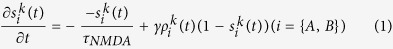



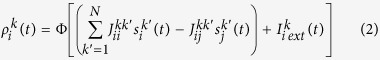



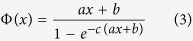



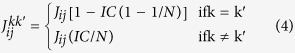


where the superscript *k* denotes the module, *s*_*i*_ is the fraction of NMDA channels open in population *i* (*i* can be either A or B), 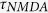 is the NMDA closure time, *γ* is a parameter of the NMDA opening rate, 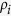 is population *I*’s firing rate, 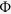 is an effective input-output relation between the synaptic input and the firing rate, 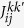 is the connection weight from population *i* in module *k* to population *j* in module 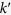. This connection weight is determined by the interconnection parameter *IC* and 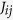 that is the connection weight between populations in the same module when the modules are taken as fully independent 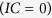. When 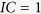, all modules are fully connected, thus all populations *i*, throughout the entire network of modules, are connected with the same weight to every population *j*, independently of the module they belong to. 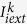 is the external synaptic current that arrives to population *i* in module *k*. 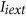 has two separate contributions, the sensory signal, that is the same for every module, and the background noise produced by synaptic bombardement that differs for every module. The latter is modeled as an Ornstein-Ulhenbeck process that arises from the background noise filtered by the AMPA channel time constant (constant background is 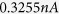, O-U time constant is 10 *ms* and O-U variance is 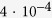 nA^2^). The connection weights are 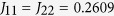 nA and 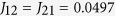 nA. The response function parameters are 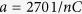, 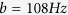 and 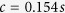.

### Behavioral task

Taken from^24^, participants fixated a central red dot (diameter of 0.56°) on a gray background (50 cd/m^2^) for 200 ms. Two flickering gray patches were presented at both sides of the fixation dot until a response was made. Patches were presented on the horizontal meridian, centered at ±1.04° from the fixation point. Each patch was composed of four vertical, spatially adjacent bars (0.14° × 0.56°). The luminance of the bars was updated synchronously every 40 ms, sampling from a Gaussian distribution with a standard deviation of 10 cd/m^2^. The mean of this distribution equaled the luminance of the background for one of the patches and was set higher for the other (referred as “target”). Subjects simultaneously reported the location they considered was the target and a binary confidence (high or low).

### Psychophysical kernels

The luminance fluctuations are pooled into four groups depending on the subject’s selection and reported confidence. The groups are: 
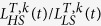
 (fluctuations of the selected patch with high/low confidence) and 
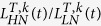
 (fluctuations of the non-selected patch with high/low confidence), where the super-index *T* indicates the trial, *k* indicates the bar within the patch, and *t* is the time. The decision and confidence psychophysical kernels are computed from these groups as:


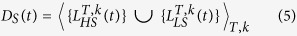



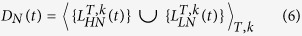



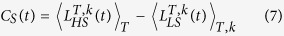



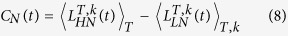


where the average is taken over trials and bars within each patch, and 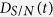 and 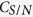 are the decision and confidence kernels respectively.

### Simulation protocol

The correlations between our model’s accuracy, RT and task discriminability were studied by simulating 2000 independent trials for several *IC*s and discriminabilities (*d*_*i*_), where a network of *N* = 100 modules was stimulated with two competing sensory signals. Both sensory signals were resampled each 40 ms from a gaussian with 5 cd/m^2^ standard deviation, and mean 50 cd/m^2^ (distractor) and 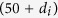 cd/m^2^ (target). The network spent 0.2 s with no stimulation before stimulus onset. The sensory signal was transformed to neural input as 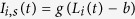, where 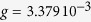 nA m^2^/cd and 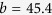 cd/*m*^2^.

The asymmetrical influence of the sensory fluctuations on an ANN’s RT were studied by performing 10000 independent simulations, where a network of N=100 modules had to decide under 2 different stimulation protocols (SP1 and SP2 from Fig. 4). In all stimulation protocols, the network had no stimulation during 0.2 s and then all stimuli were turned on. The baseline sensory input was 50 cd/m^2^. In SP 1, input targeting A had a positive fluctuation of 1 cd/m^2^ during the first 40 ms. In SP 2, input targeting B a had a negative fluctuation of 1 cd/m^2^ during the first 40 ms.

### Data fitting

To fit the behavioral data, sensory input, that represented the average patch luminance observed by the subjects, was sent to the network of 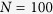 modules. The onset of the stimulation was after a 1 s wait period. The mean patch luminance was resampled each 40 ms from a gaussian distribution with 5 cd/m^2^ standard deviation, and mean value that changed over trials. The mean target luminance was taken from the distribution of mean luminances observed by the subjects, and the mean distractor luminance was fixed at 50 cd/m^2^. The network was forced to decide within 1 s after stimulus presentation, and penalized for non-decided trials, and early decision (prior to stimuli presentation). The sensory input was transformed into neural input by a linear transformation as 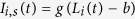. The parameters *g* and *b* were determined by fitting subjects’ decision kernel and task performance, using a covariance matrix adaptation evolutionary strategy CMA-ES. The algorithm efficiently explores the parameter space in order to find the parameter values that minimize a merit function. The implementation was taken from^59^. The merit function we used was:


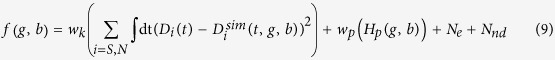


where the first term is the squared difference between the subject and model’s decision kernels, 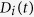 and 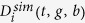 respectively. In the second term, 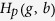 is the Pearson chi squared test statistic^60^ that the subject and simulation’s number of hits and misses come from the same multinomial. 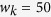 is the weight of the least squares and 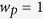 the weight of the Pearson statistic. The merit function is penalized by the number of early decision trials (*N*_*e*_, decisions prior to stimulus onset) and the number of non-decided trials (*N*_*d*_, trials where no option was selected). These forced the network to commit to a choice only due to the sensory input in 1 s. The model’s decision kernel and task accuracy were approximated by simulating 10000 trials for each parameter set. Each trial’s mean target luminance was taken from the distribution of mean target luminances observed by all subjects. The values of *g* and *b* that minimized the merit function were 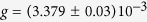
nA m^2^/cd and 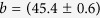
cd/m^2^.

Once the decision kernel and performance were fitted using the luminance transformation parameters, the confidence report rate (number of high confidence hits and misses, and low confidence hits and misses) was fitted. We propose that a separate neural layer decodes confidence from the estimate of *σ*_*dv*_. The dispersion was estimated with the activity of pools CA/CB (the equivalent to FMC but computed with neural populations as shown in SI sec.B) at decision time. The probability of high confidence is taken as 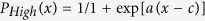, where *x* is the dispersion’s estimate. We determine *a* and *c* by sampling the model’s binary confidence responses from 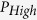 and minimizing the Pearson chi squared test statistic between the model and subject’s performance discriminated by confidence (number of high-hits, high-misses, low-hits and low-misses). Again, we used the same CMA-ES optimization algorithm to perform these fits^59^. The resulting parameters were, 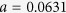 s and 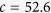 Hz. The results are qualitatively the same when taking the artificially counted FMC as the estimate of *σ*_*dv*_ at response time.

## Additional Information

**How to cite this article**: Paz, L. *et al.* Confidence through consensus: a neural mechanism for uncertainty monitoring. *Sci. Rep.* **6**, 21830; doi: 10.1038/srep21830 (2016).

## Supplementary Material

Supplementary Information
