# Supplementary material for: Confidence through consensus: a neural mechanism for uncertainty monitoring
Source: Sci Rep. 2016 Feb 24;6:21830. doi: 10.1038/srep21830 (PMC4764837; doi:10.1038/srep21830)
Supplement: Supplementary Information [file srep21830-s1.pdf]

# Supplementary Information. Confidence through consensus: a neural mechanism for uncertainty monitoring

**Luciano Paz[1,*], Andrea Insabato[2], Ariel Zylberberg[3], Gustavo Deco[2], and Mariano Sigman[1,4]**

[1]Integrative Neuroscience Laboratory, IFIBA, CONICET and Physics Department, FCEyN, UBA, Buenos Aires, Argentina
[2]Universidad Pompeu Fabra, Barcelona, Spain
[3]Department of Neuroscience, Howard Hughes Medical Institute, Columbia University, New York, NY 10032
[4]Universidad Torcuato di Tella, Buenos Aires, Argentina
[*]lpaz@df.uba.ar

## ABSTRACT

Diffusion models to a threshold can explain with great precision performance and response time distributions in binary decisions. One challenging problem for decision models is to account for the relation between decision time and confidence. During the decision formation, ramping neurons represent evidence and it is assumed that time should be estimated in separate dedicated circuits. Here we explain how decision time and confidence can be read-out from the activity of neurons that integrate evidence during decision making. We propose a simple mechanism based on basic theoretical principles. In a diffusion process, time and variability are correlated. Hence, measuring the variance of firing rates at the moment of the decision provides a natural way to measure response time. This, in turn can be used to compute confidence. Here we show that in fact confidence can be read-out from the ensemble variability of a pool of weakly-connected decision neurons. We explicitly construct a neuronal architecture capable of estimating time, and show that this model can explain subtle empirical findings of the relation of time and confidence in human decision making experiments. Our results also make concrete predictions on how population activity of decision neurons should relate to response time and confidence.

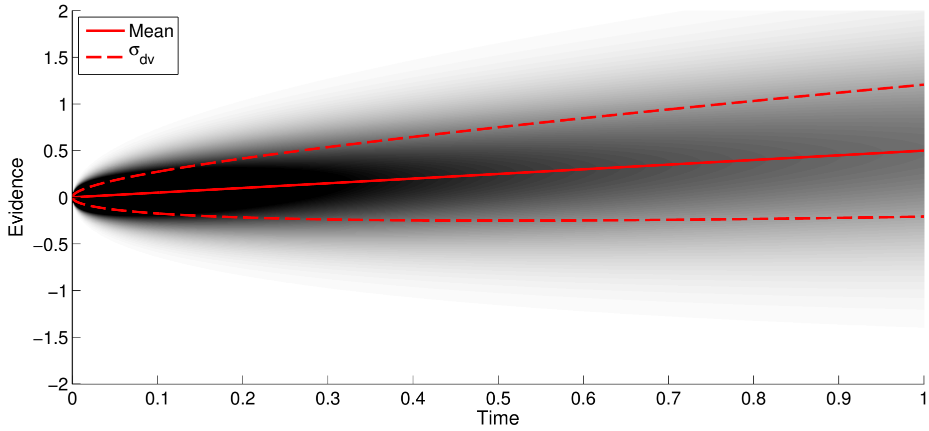


**Figure S1.** Probability density of the evidence accumulated with a drift diffusion process as a function of time. The mean and the enveloping standard deviation are plotted as solid and dashed lines respectively.

## A Variance of the state of a diffusion process depends on time

The temporal evolution of variables corrupted by noise is described by stochastic dynamical equations of the form:

$$\frac{dx}{dt} = a(x,t) + b(x,t)\xi(t) \tag{1}$$

where $a$ and $b$ are the deterministic functions and $\xi$ is a stochastic variable. Depending on the stochastic force's (also called noise) distribution, the probability density $p(x,t|x_0,t_0)$ evolves differently. In particular, if $\xi(t)$ is normally distributed with independent samples at different times, the probability density evolves following the Fokker-Planck master equation

$$\frac{\partial p(\mathbf{z},t|\mathbf{y},t')}{\partial t} = -\sum_i \frac{\partial}{\partial z_i}\left[A_i(\mathbf{z},t)p(\mathbf{z},t|\mathbf{y},t')\right] + \sum_{i,j}\frac{1}{2}\frac{\partial^2}{\partial z_i \partial z_j}\left[B_{ij}(\mathbf{z},t)p(\mathbf{z},t|\mathbf{y},t')\right] \tag{2}$$

where $A$ controls drift and $B$ diffusion. The case without drift and constant diffusion is simply a Wiener process. The probability density $p(x,t|x_0,t_0)$ takes the form of a gaussian distribution with variance proportional to $t-t_0$. Adding a constant drift does not change the variance's evolution (Fig. S1). If $A$ or $B$ depend on $x$, the probability density follows other dynamics such as Ornstein-Ulhenbeck, or non-linear diffusion. Both of these present correlations between elapsed time and the probability distribution's dispersion. For a detailed deduction refer to[1] chapter 3.

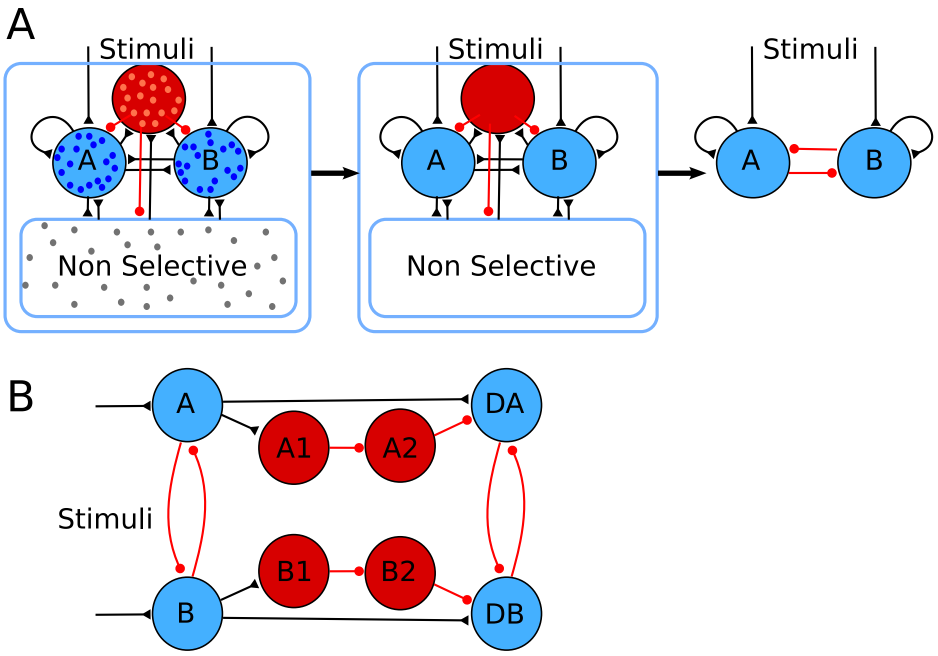


**Figure S2.** A. Neural perceptual decision network sketch for a single module from[3]. Populations are divided into excitatory selective (blue), excitatory non selective (white) and inhibitory (red). The progression shows the dimensionally reduced meanfield model. B. Firing rate threshold crossing detection mechanism from[7]. Populations A and B compete, when one crosses a threshold of activity the first layer of interneurons is activated. This first layer inhibits the second layer and thus releases population DA or DB in order for it to become active. Note that DA and DB receive direct connections from the competing layer whilst we propose another mechanism for DA and DB to become active once A and B surpass the threshold. The interneuron populations that mediate the detection are proposed to be in the Caudate Nucleus and the Superior Colliculus in the Basal Ganglia.

## B Neural implementation

We base our neural decision model on an extensively studied model of perceptual decision making in which four populations of neurons interact[2]. Two pools of pyramidal neurons are sensitive to the sensory input of the decision process, while the others, one pool of pyramidal neurons and one pool of interneurons, are non selective. All pools are fully connected with each other through AMPA, NMDA and $GABA_A$ mediated synapses (Fig. S2.A). The pyramidal neurons outward connections target AMPA and NMDA receptors while the interneurons target $GABA_A$. Furthermore, all pools of neurons receive background Poisson spike trains that target AMPA receptors and yield a baseline of synaptic input current. The slow NMDA channel dynamics, along with balanced inhibition and positive recurrent connections, lead the whole system to a winner take all state where one of the selective pools of neurons becomes active and the other is inhibited. The network of single neurons and their synapses can be reduced to an effective two-dimensional meanfield model where the fractions of open NMDA channels drive the decision process[3,4] (Fig. S2.A). The complete deduction can be found in[3]. Note that the four populations with three neurotransmitter channel dynamics were reduced to two populations with a single effective NMDA channel opening dynamic. Thus these populations are a mixture of pyramidal and interneurons, and as such follow an altered input-output relation (i.e. output firing rate as a function of input current). Furthermore, the background synaptic input, that is a vital part of the model, is included in the meanfield as dynamic Ornstein-Ulhenbeck process[5] plus a fixed value. In the numerical simulations, an exact update method is used to sample the Ornstein-Ulhenbeck noise (Refer to Eq. E-12b in Gillespie 1992[6]).

If after the stimulus presentation, population 1 has increased firing rate while 2 has baseline activity, then option 1 is selected. The firing rate of each population can be interpreted as the accumulated evidence in favor of each option, and the selection takes place after an activity threshold is crossed. The threshold crossing can be signaled by a separate bursting population that only fires once one of the competing populations surpasses a certain level of activity. Lo and Wang[7] proposed a possible implementation, schematically shown in Fig. S2.B. In Lo's implementation, populations A and B integrate sensory evidence and compete with each other. If, for example, A surpasses a threshold of activity, it activates population A1. A1 in turn inhibits A2, which is normally active due to background input. As A2 is silenced, inhibition on DA is released, allowing the direct connection from A to produce a burst of activity in DA. We use a simplified implementation, where DA and DB become active due to background input, and not by direct connections from the competition layer, once the inhibition is released.

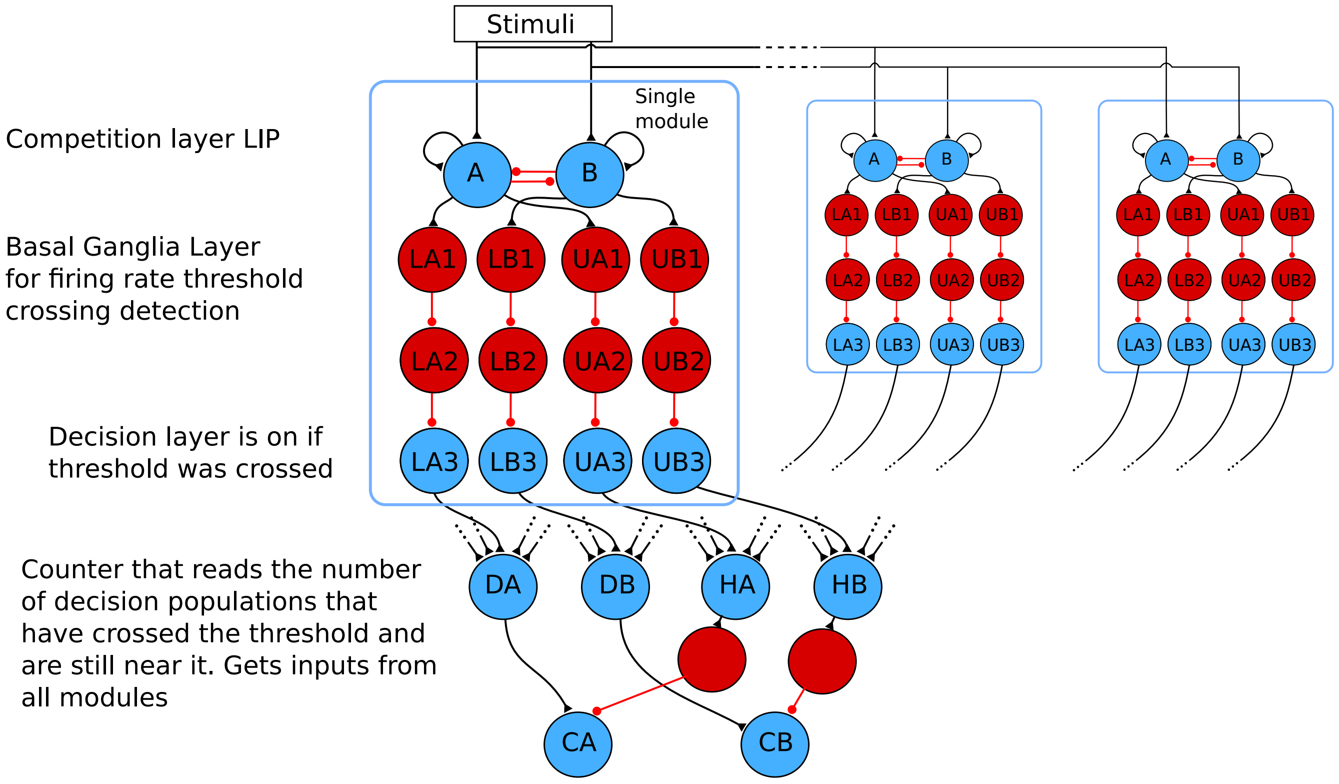


**Figure S3.** Extended neuronal implementation with the competition (A and B), threshold crossing detection (L and U) and counting layers (D, H and C). All modules are interconnected although the connections are not depicted here. We propose that all populations receive different levels of background synaptic input. This background allows the second layer of the modules to have high baseline activity, and also allows the third layer of each module to have high activity once the inhibition from the second layer disappears.

We propose an ensemble network model of $N = 100$ modules of these decision pools that are weakly connected with each other. A crucial aspect of the model is how it determines the selected option. In the single module case, each population's firing rate could be interpreted as the evidence in favor of an alternative. In the multi module case, the mean across populations $\langle \rho_i^k(t) \rangle_k$ could serve as the evidence in favor of option $i$. It is presently unclear how this computation could take place but there is evidence supporting normalization as a possible neural computation[8]. Alternatively, the decision could be signaled after over half the modules have crossed a threshold of activity. The simulations shown in the main text use the latter criterion but both criteria yield almost the same results.

The neural implementation is divided into:

1. The competition layer
2. The threshold crossing detection layer
3. The counting layer

The competition layer is made up by the neural populations that receive sensory input and compete in an attractor neural network (ANN) fashion (Fig. 1.A and Fig. S2.A). This layer is the heart of the entire decision model. The activity of each population can be artificially read (i.e. not using a neural mechanism) in order to detect the selected option and the inter module dispersion, $\sigma_{dv}$. We go further by addressing the problem of "how could the decision be detected and $\sigma_{dv}$ be read with neurons?". This is done by extending the competition layer with the threshold crossing detection and the counting layers (Fig. S3).

The detailed dynamics that the competition layer obey are detailed in table 1.

**Table 1.** The competition layer

| A | Model Summary |
|---|---|
| **Populations** | N identical modules with two populations, A and B, each. $A^k$ is module's $k$, A population |
| **Topology** | Excitatory recurrent connections and inhibitory lateral connections within modules. Modules are interconnected with excitatory (inhibitory) connections if one each module's population is the of the same (different) kind, i.e A with A or B with B. |
| **Connectivity** | — |
| **Neuron model** | Meanfield rate model governed by a fixed synaptic input - firing rate output relation. |
| **Channel models** | Instantaneous AMPA and $GABA_A$ channel dynamics (i.e. fraction of channels open proportional to presynaptic firing rate). Detailed NMDA channel dynamics. |
| **Synapse model** | Continuous current dependent on the fraction of NMDA channels open or the firing rate, if an interneuron. |
| **Plasticity** | — |
| **Input** | Two sensory stimuli, one given to all A populations and the other to all B populations. Background synaptic bombardment given by a constant current plus an Ornstein-Ulhenbeck process |
| **Measurements** | Firing rate, number of populations A and B that crossed an activity threshold, inter module STD, number of populations A and B with firing rate between two levels of activity |

| B | Populations | |
|---|---|---|
| **Name** | **Elements** | **Size** |
| A | Meanfield competition approximation | 1 per module |
| B | Meanfield competition approximation | 1 per module |

| C | | | Connectivity |
|---|---|---|---|
| **Name** | **Source** | **Target** | **Pattern** |
| AA | $A^k$ | $A^{k'}$ | weight $0.2609(1-IC(1-1/N))$ if $k=k'$, $0.2609(IC/N)$ otherwise |
| BB | $B^k$ | $B^{k'}$ | weight $0.2609(1-IC(1-1/N))$ if $k=k'$, $0.2609(IC/N)$ otherwise |
| AB | $A^k$ | $B^{k'}$ | weight $-0.0497(1-IC(1-1/N))$ if $k=k'$, $-0.0497(IC/N)$ otherwise |
| BA | $B^k$ | $A^{k'}$ | weight $-0.0497(1-IC(1-1/N))$ if $k=k'$, $-0.0497(IC/N)$ otherwise |

| D | Neuron and Synapse Model |
|---|---|
| **Name** | Meanfield competition approximation |
| **Type** | Firing rate, NMDA channel |
| **Dynamic equations** | $\frac{\partial s_i^k(t)}{\partial t} = -\frac{-s_i^k(t)}{\tau_{NMDA}} + \gamma \rho_i^k(t)\left(1-s_i^k(t)\right)$ <br> $\rho_i^k(t) = \Phi\left[\left(\sum_{k'=1}^{N} J_{ii}^{kk'} s_i^{k'}(t) - J_{ij}^{kk'} s_j^{k'}(t)\right) + I_{iext}^k(t)\right]$ <br> $\Phi(x) = \frac{270\,1/nC\,x + 108Hz}{1-\exp\left[-0.154s\,(270\,1/nC\,x + 108Hz)\right]}$ |

| E | Input |
|---|---|
| **Dynamic equations** | $I^k_{iext}(t) = I^k_{istim}(t) + I_{Background} + I^k_{inoise}(t)$<br>$I_{Background} = 0.3255nA$<br>$I^k_{inoise}(t+dt) = (1-dt/\tau_{noise})I^k_{inoise}(t) + \eta(t)\sqrt{0.5*(1-exp(2dt/\tau_{noise}))}0.02nA$ |

**Table 2.** Threshold crossing detection layer

| A | Model Summary |
|---|---|
| **Populations** | N independent modules. Populations L1(A/B), L2(A/B), L3(A/B) and U1(A/B), U2(A/B), U3(A/B) |
| **Topology** | Independent branches to deeper layers. |
| **Connectivity** | — |
| **Neuron model** | Meanfield rate model governed by a fixed synaptic input - firing rate output relation. |
| **Channel models** | Instantaneous AMPA and $GABA_A$ channel dynamics (i.e. fraction of channels open proportional to presynaptic firing rate). Detailed NMDA channel dynamics. |
| **Synapse model** | Continuous current dependent on the fraction of NMDA channels open or, if in the first two layers, the presynaptic firing rate. |
| **Plasticity** | — |
| **Input** | Output from populations $A^k$ and $B^k$ reach the first layer. The second and third layer receive background synaptic bombardment. |
| **Measurements** | — |

| B | Populations | |
|---|---|---|
| **Name** | **Elements** | **Size** |
| L1(A/B), L2(A/B), U1(A/B), U2(A/B) | Interneuron meanfield approximation | 1 per module |
| L3(A/B) and U3(A/B) | Pyramidal neuron meanfield approximation | 1 per module |

| C | Connectivity | | |
|---|---|---|---|
| **Name** | **Source** | **Target** | **Pattern** |
| competition to L1 | $A^k$ ($B^k$) | $L1A^k$ ($L1B^k$) | weight $J_L = I_{th}(1 + 1/(15\text{Hz}\gamma\tau_{NMDA}))$ |
| competition to U1 | $A^k$ ($B^k$) | $U1A^k$ ($U1B^k$) | weight $J_U = I_{th}(1 + 1/(20\text{Hz}\gamma\tau_{NMDA}))$ |
| first to second | $L1A^k$ (or any population in layer 1) | $L2A^k$ (corresponding population in layer 2) | weight $J_I = 0.07nC$ |
| second to third | $L2A^k$ (or any population in layer 2) | $L3A^k$ (corresponding population in layer 3) | weight $J_I = 0.07nC$ |

| D | Neuron and Synapse Model |
|---|---|
| **Name** | Interneuron and pyramidal meanfield approximation |
| **Type** | Firing rate, instantaneous GABA channel |
| **Dynamic equations** | $\rho_i^k(t) = \Phi\left(I_{inet}^k(t) + I_{iext}^k(t)\right)$ <br> $\Phi(x) = \frac{615\,1/nCx + 177Hz}{1-\exp[-0.087s(615\,1/nCx + 177Hz)]}$ if interneuron <br> $\Phi(x) = \frac{310\,1/nCx + 125Hz}{1-\exp[-0.16s(310\,1/nCx + 125Hz)]}$ if pyramidal neuron <br> $I_{inet}^k(t) = \begin{cases} J_{L/U}s_A^k(t) & \text{if } i \text{ is L1A}^k \text{ or U1A}^k \\ J_{L/U}s_B^k(t) & \text{if } i \text{ is L1B}^k \text{ or U1B}^k \\ J_I\rho_{i-1}^k(t) & \text{if } i \text{ is layer 2 or 3 population} \end{cases}$ |

| E | Input |
|---|---|
| **Dynamic equations** | $I_{iext}^k(t) = I_{Background} + I_{inoise}^k(t)$ <br> $I_{Background} = \begin{cases} 0nA & \text{if population in layer 1} \\ 0.416nA & \text{if population in layer 2} \\ 0.9nA & \text{if population in layer 3} \end{cases}$ <br> $I_{inoise}^k(t+dt) = (1-dt/\tau_{noise})I_{inoise}^k(t) + \eta(t)\sqrt{0.5*(1-exp(2dt/\tau_{noise}))}\sigma_i$ <br> $\sigma_i = 0.002nA$ if $i$ is an interneuron <br> $\sigma_i = 0.01nA$ if $i$ is an pyramidal neuron |

**Table 3.** Counting layer

| A | Model Summary |
|---|---|
| **Populations** | 6 populations DA, DB, HA, HB, CA and CB |
| **Topology** | — |
| **Connectivity** | — |
| **Neuron model** | Meanfield rate model governed by a fixed synaptic input - firing rate output relation. |
| **Channel models** | Instantaneous AMPA and $GABA_A$ channel dynamics (i.e. fraction of channels open proportional to firing rate). |
| **Synapse model** | Continuous current dependent on the firing rate. |
| **Plasticity** | — |
| **Input** | Output from $LA3^k$, $LB3^k$, $UA3^k$ and $UB3^k$. Background synaptic bombardment given by a constant current plus an Ornstein-Ulhenbeck process |
| **Measurements** | Firing rate. |

| B | Populations | |
|---|---|---|
| **Name** | **Elements** | **Size** |
| DA | Pyramidal neuron meanfield approximation | 1 |
| DB | Pyramidal neuron meanfield approximation | 1 |
| HA | Pyramidal neuron meanfield approximation | 1 |
| HB | Pyramidal neuron meanfield approximation | 1 |
| CA | Pyramidal neuron meanfield approximation | 1 |
| CB | Pyramidal neuron meanfield approximation | 1 |

| C | Connectivity | | |
|---|---|---|---|
| **Name** | **Source** | **Target** | **Pattern** |
| layer 3 to D | L3A/B$^k$ | DA/B | weight $J_D = 0.68/N$ |
| layer 3 to H | U3A/B$^k$ | HA/B | weight $J_D = 0.68/N$ |
| D and H to C | DA/B and HA/B | CA/B | weight $J_C = 0.0032$ |

| D | Neuron and Synapse Model |
|---|---|
| **Name** | Meanfield competition approximation |
| **Type** | Firing rate, instantaneous AMPA and $\mathrm{GABA_A}$ |
| **Dynamic equations** | $\rho_i(t) = \Phi\left(I_{inet}(t) + I_{i\,ext}(t)\right)$ <br> $\Phi(x) = \dfrac{310\,1/nC\,x + 125Hz}{1 - \exp\left[-0.16s\,(310\,1/nC\,x + 125Hz)\right]}$ <br> $I_{inet}(t) = \begin{cases} \sum_k J_D s^k_{\mathrm{LA3/LB3}}(t) & \text{if } i \text{ is DA/B} \\ \sum_k J_D s^k_{\mathrm{UA3/UB3}}(t) & \text{if } i \text{ is HA/B} \\ J_C(\rho_{\mathrm{DA/B}}(t) - \rho_{\mathrm{HA/B}}(t)) & \text{if } i \text{ is CA/B} \end{cases}$ |

| E | Input |
|---|---|
| **Dynamic equations** | $I_{i\,ext}(t) = I_{Background} + I^k_{inoise}(t)$ <br> $I_{Background} = 0.416nA$ <br> $I^k_{inoise}(t+dt) = (1 - dt/\tau_{noise})I^k_{inoise}(t) + \eta(t)\sqrt{0.5 * (1 - exp(2dt/\tau_{noise}))}0.01nA$ |

The threshold crossing detection and counting layer follow similar dynamics. The populations in each layer are meanfield approximations of a groups of integrate and fire neurons. However, the neurons inside each population are either pyramidal or interneurons, and not mixtures as in the competition layer. Thus, the input-output relation and the synapses' dynamics change. The detailed description can be found in tables 2 and 3. The main synaptic differences are that:

1. The interneurons only target GABA receptors. The GABA channel dynamics is approximated as instantaneous, thus the postsynaptic current is proportional to the interneuron's firing rate.

2. The counting layer's excitatory connections only are mediated by AMPA. This implies that, as with the interneurons, the postsynaptic current is proportional to the presynaptic firing rate.

The proposed threshold crossing detection layer is based on the mechanism proposed by Lo and Wang[7] to detect when a neural population surpasses a given firing rate value. It relies on the widely used mechanism of disinhibition[9–13]. Each

decision module in Fig. 1 .A is connected to a module that detects when one of the two decision pools crosses the threshold for the decision, as shown in Fig. S3. The threshold module is composed of three layers and each layer has two branches, L and U. Branch L is needed to signal the crossing of the lower decision threshold $\lambda$, while branch U is needed to detect whether the module is in a given interval near the threshold or already crossed the upper threshold $\lambda + \Delta\lambda$. The decision threshold mechanism works as following. The second and third layer receive background input that would drive them to spike at a constant firing rate. However, the second layer constantly inhibits the third layer so it is silent. When one of the two decision pools (e.g. A) increases the activity, due to the decision process, and crosses the decision threshold (15Hz), the corresponding pool from layer one (LA1) of the threshold module is activated. This pool, in turn, inhibits its corresponding layer 2 population (LA2), thus releasing inhibition from the corresponding third layer (LA3). Hence LA3 becomes active due to the background input if A crosses the decision threshold. This implies that the third layer works as a binary indicator. It is firing ("on") if the competition population surpassed a certain threshold and is silent ("off") otherwise. The upper threshold mechanism (branch U) works in the same way but its threshold is set to a slightly higher value (20Hz).

The global pools DA, DB, HA and HB receive input from all modules, thereby counting how many modules crossed the lower (DA and DB) and the upper threshold (HA and HB). The final commitment to a choice is based on the activity of pools DA and DB. When DA or DB surpass a certain level of activity that corresponds to over half the modules having committed to the same decision (97.68Hz for the parameters we used), the decision is taken. In order to form a confidence judgment the number of modules near the threshold (FMC) is estimated by the confidence pools (CA and CB) that calculate the difference between the activity of LA and HA for decision A (or LB and HB for decision B). The firing rate CA, if option A is selected, is used as input to a sigmoid that determines the probability of high confidence as, $P_{High}(x) = 1/1 + \exp[a(x-c)]$. The parameters of the sigmoid control the bias and the slope of the transition from high to low confidence, and were determined by fitting the subjects' accuracy discriminated by confidence.

### B.1 Description of model dynamics

An example of the network's dynamics during a single trial is shown in fig. S4. During the shown trial, the target's mean luminance was set to $52cd/m^2$ and the distractor was set to $50cd/m^2$. The A populations were sensitive to the target patch while the B populations were sensitive to the distractor. During the presented simulation, $IC = 0$.

The competing layer integrates sensory evidence for each option. A single module's pair of competing $A^k$ and $B^k$ populations is shown (Fig. S4.A). These competing populations drive the firing rates of the first layers of the threshold detection portion of the network (Fig. S4.B). These, in turn, release the inhibition placed on the third layer LA3, LB3, UA3 and UB3 (Fig. S4C). This layer works as a binary indicator value. The counting layer simply counts the number of third layer populations active (Fig. S4.D).

Populations LA1, LB1, UA1 and UB1 receive synaptic input from competing neurons in each module, and become "active" (fire sufficient spikes in order to inhibit populations LA2, LB2, UA2 or UB2) after receiving more than $I_{th}$ current. The value of $I_{th}$ is determined in order to have a correct count method.

When population A has a constant firing rate, $\rho_A$, its NMDA gating variable is at a steady state, $s_A = (1 + 1/(\rho_A \gamma \tau_{NMDA}))^{-1}$. The synaptic current sent to populations LA1 and UA1 will be equal to

$$I_{L/U} = w_{L/U} \left(1 + \frac{1}{\rho_A \gamma \tau_{NMDA}}\right)^{-1} \tag{3}$$

where $w_{L/U}$ is the connection weight to populations LA1 or UA1. Assuming a value of $I_{th}$, the connection weights can be calculated in order to detect the crossing at a given firing rate value $\rho_A$. $w_L = I_{th}(1 + 1/(15\text{Hz}\gamma\tau_{NMDA}))$ and $w_L = I_{th}(1 + 1/(20\text{Hz}\gamma\tau_{NMDA}))$. The best value for $I_{th}$ is the one that yields the best linear dependence between the number of competing populations that have crossed the threshold and the firing rates of DA, DB, HA and HB. However, synaptic noise in the threshold crossing detection and counter layers induce variability that makes measuring the best $I_{th}$ value complicated. In order to measure $I_{th}$, we perform simulations fixing the noise to zero in the threshold crossing detection and counter layers (Fig. S5). The observed best linear dependence occurs at $I_{th} = 0.29$nA.

The resulting network yields the same behavior as the model with artificial counting.

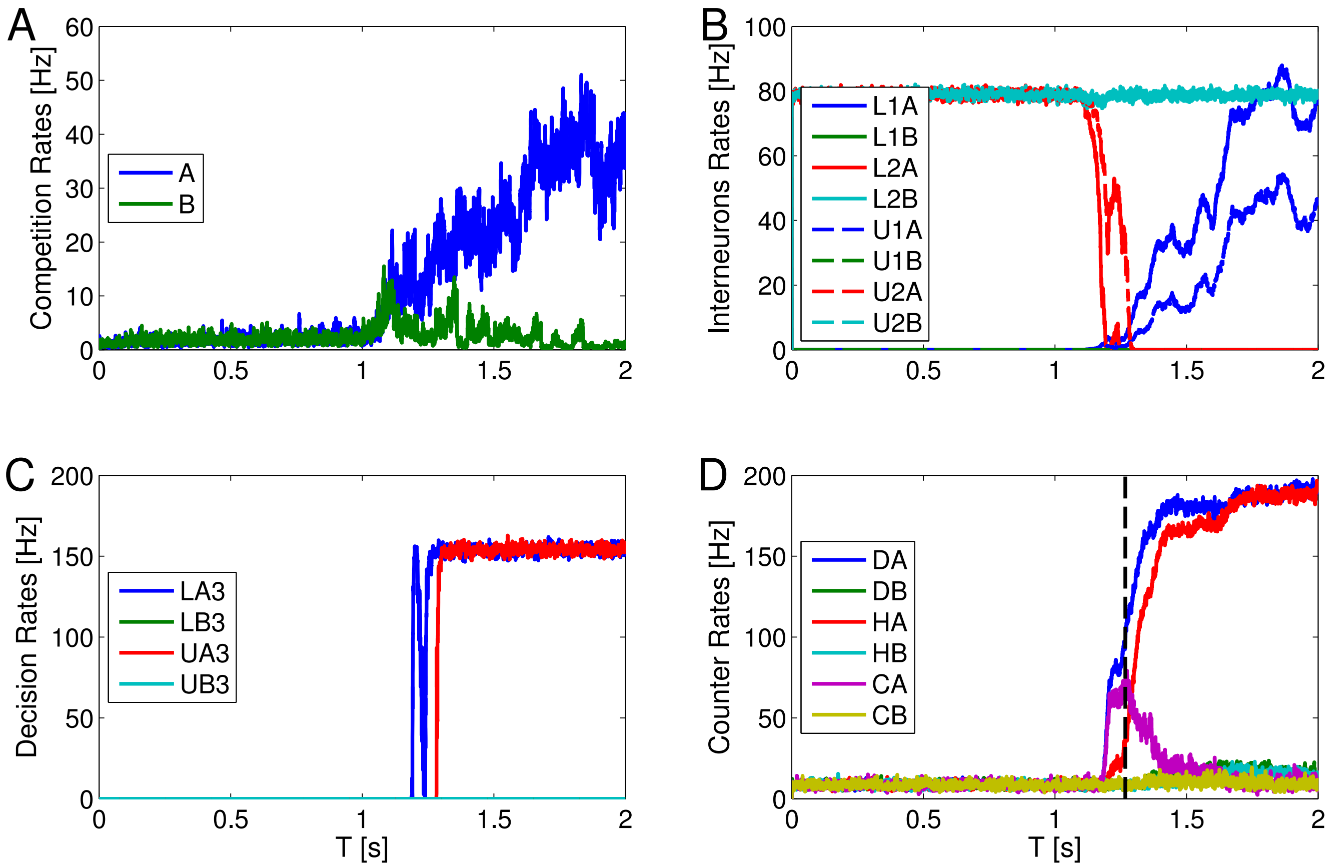


**Figure S4.** A single trial is simulated. Panels A, B and C show the firing rates of the populations in the same, single module out of 100 that were used in the decision model. A. The single module's populations A and B firing rate. In this particular module, population A wins the competition. B. The two layers of interneurons in the threshold crossing detection layers. C. Indicator populations LA3, LB3, UA3 and UB3. D. Populations D, H and C for both alternatives. Note that these are not for a single module but for the entire network. Dashed vertical black line indicates the time at which DA passed the decision threshold and option A was selected.

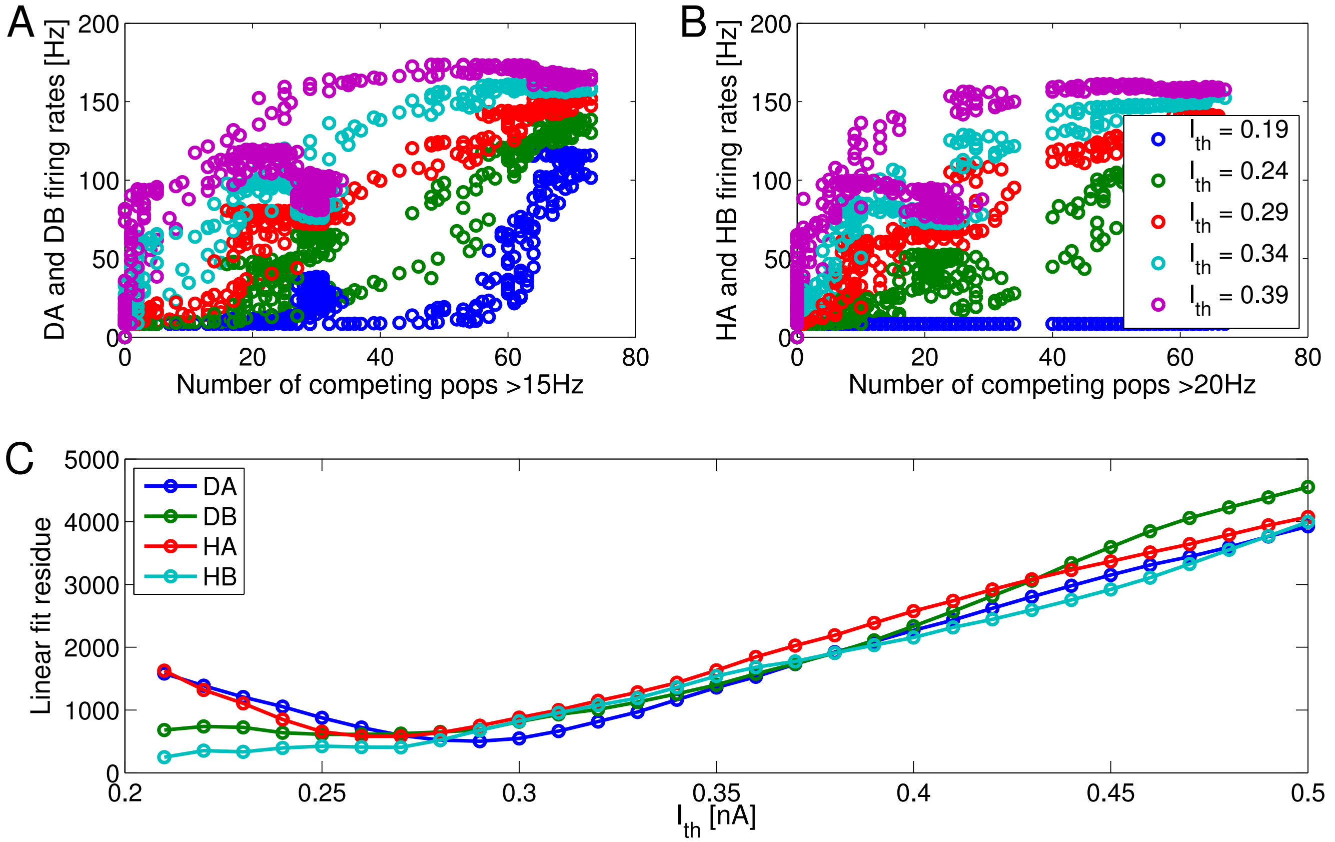


**Figure S5.** Linear dependence between artificial and neural count methods. A. Populations DA and DB firing rate as a function of the number of populations A and B with a firing rate greater than 15Hz. Data points are the firing rates and number of populations counted at each time step of a single trial's simulation. B. The same as A but with populations HA and HB, and firing rates greater than 20Hz. C. Linear fit residue as a function of $I_{th}$ values. For very small $I_{th}$, DA, DB, HA and HB remain silent at all time, leading to a good linear agreement with zero slope. Thus $I_{th} < 0.21$nA were disregarded.

## C Detailed correlations within discriminability

In Figures 2 and 3 of the main text we show correlations between our model's RT, and $\sigma_{dv}$ and FMC. These correlations were shown for averaged RT, $\sigma_{dv}$ and FMC values. The averages were done for separate discriminabilities ($d_i$) between the target and distractor patches. Each average was taken on 2000 simulated trials. However, the shown correlation between RT and $\sigma_{dv}$, and $\sigma_{dv}$ and FMC still hold within the same discriminability (Fig. S6 and S7 respectively). It is clear that even for the same discriminibability, our model's dispersion is strongly correlated with the RT, and FMC is a valid estimate for said dispersion.

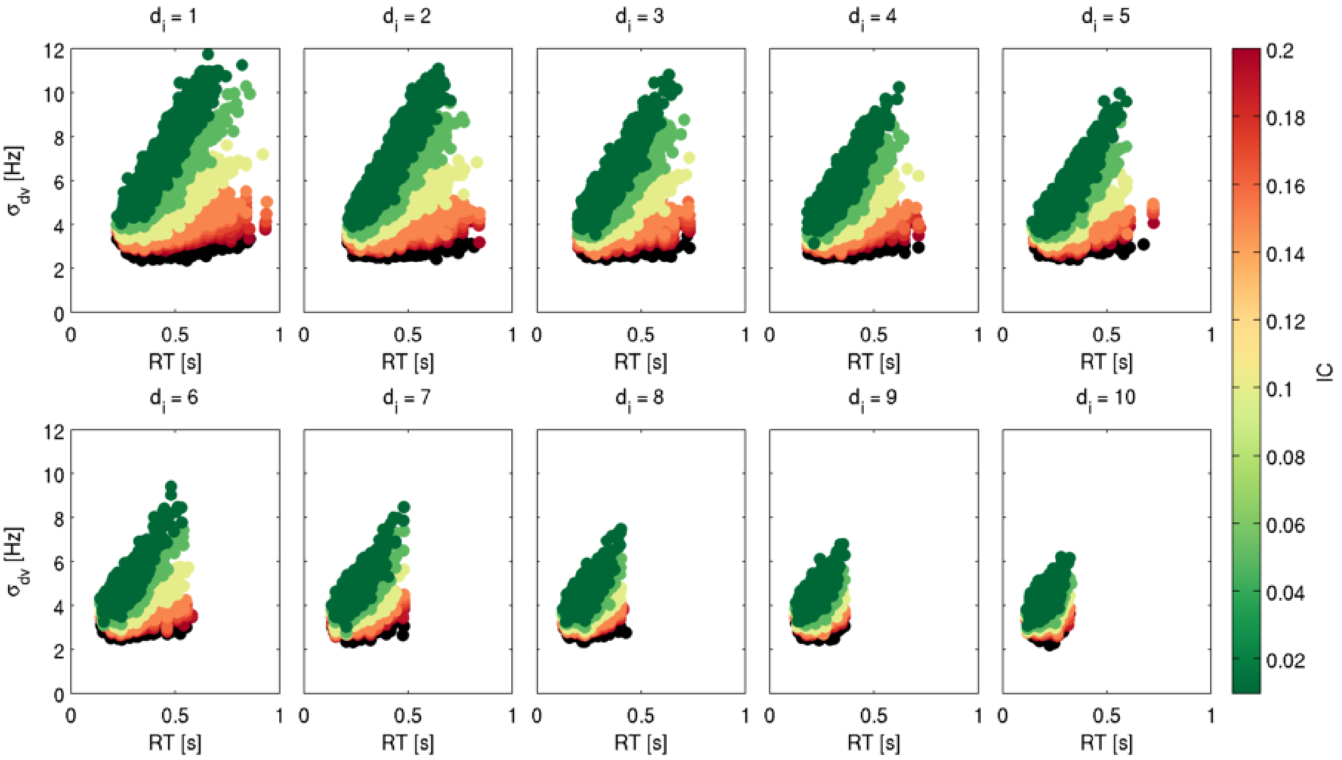


**Figure S6.** $\sigma_{dv}$ as a function of RT for different stimuli discriminibabilities ($d_i$ in cd/m$^2$), and $IC$ values. The $IC$ is encoded in the color as shown in the lateral colorbar. Black corresponds to $IC = 0.5$.

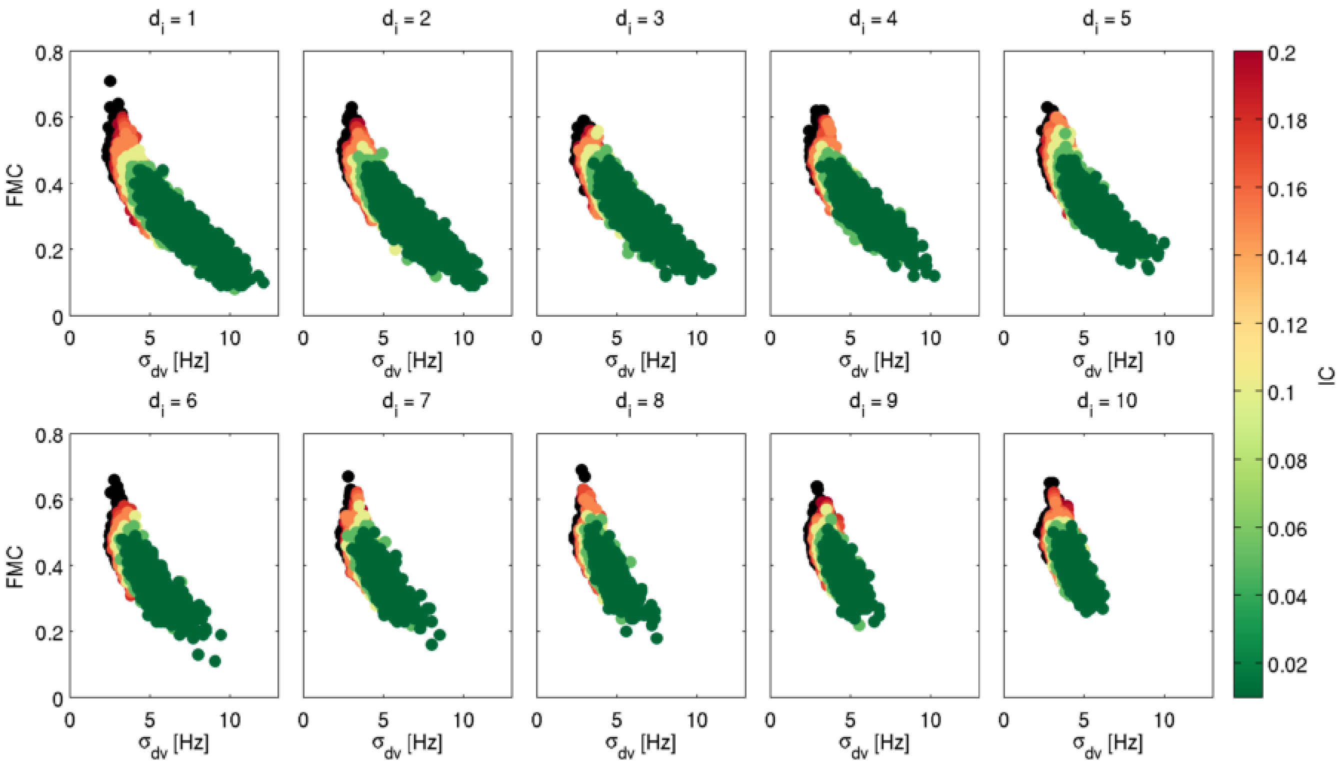


**Figure S7.** FMC as a function of $\sigma_{dv}$ for different stimuli discriminibabilities ($d_i$ in cd/m$^2$), and $IC$ values. The $IC$ is encoded in the color as shown in the lateral colorbar. Black corresponds to $IC = 0.5$.

## D Response time distribution overlap

Its important to clarify that our model does not explicitly rely on response time to compute confidence. Confidence is readout from an estimate of dispersion, which is only correlated with RT. In fact, if subjects only relied on RT to determine confidence, the distributions would not have a large overlap. However, we observe, both for simulations and for subjects' data, that RT distributions split by confidence are very overlapped, and that low confidence reports are usually associated to longer RTs (Fig. S8). The simulation's RT distribution is nothing like the subjects' distribution which in fact is expected, as we force the model to decide in less than 1s. This was done because, in this work, we are not interested in reproducing the detailed RT distribution. For our analyses it was sufficient to only look at the task accuracy and sensory fluctuation's influence. To be able to account for the RT distribution, which is by its self a very difficult task, the merit function described in section "Data Fitting" of the main text should be adapted to include the RT's goodness of fit and probably the background input or network connection weights should be changed. We deemed this was too computationally expensive and beyond our intended analysis.

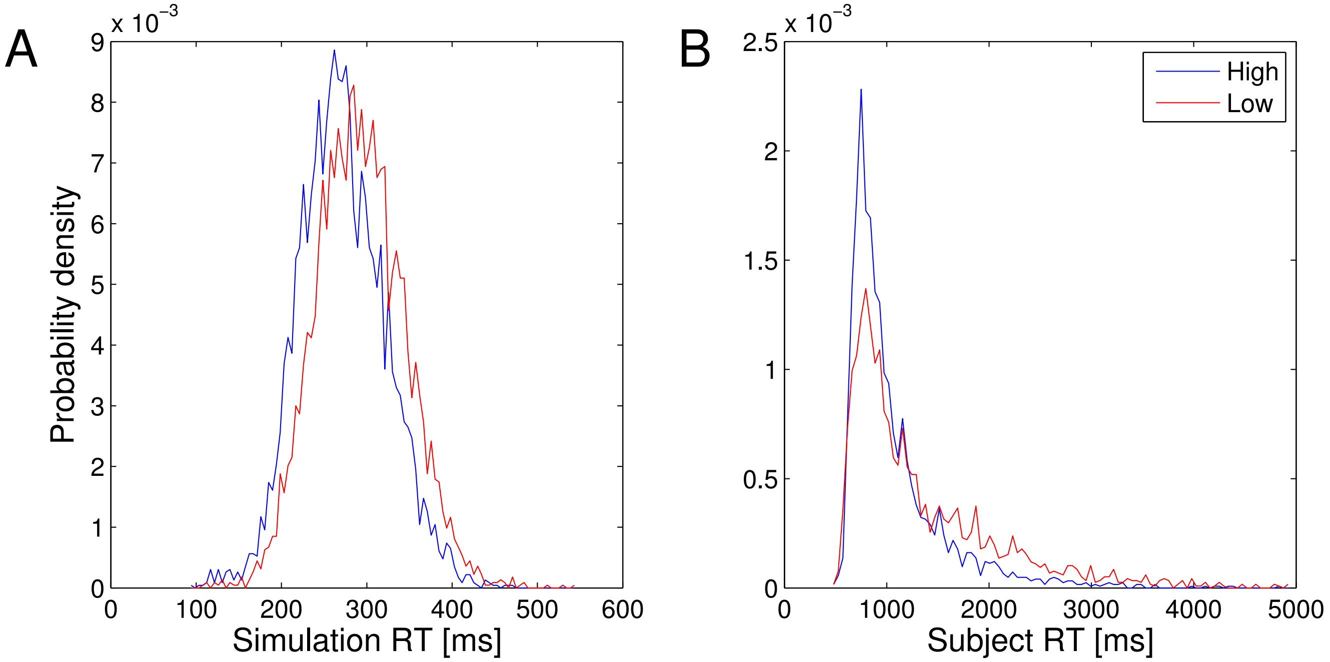


**Figure S8.** A. Simulation and B. subjects' RT distributions grouped by the resulting confidence.

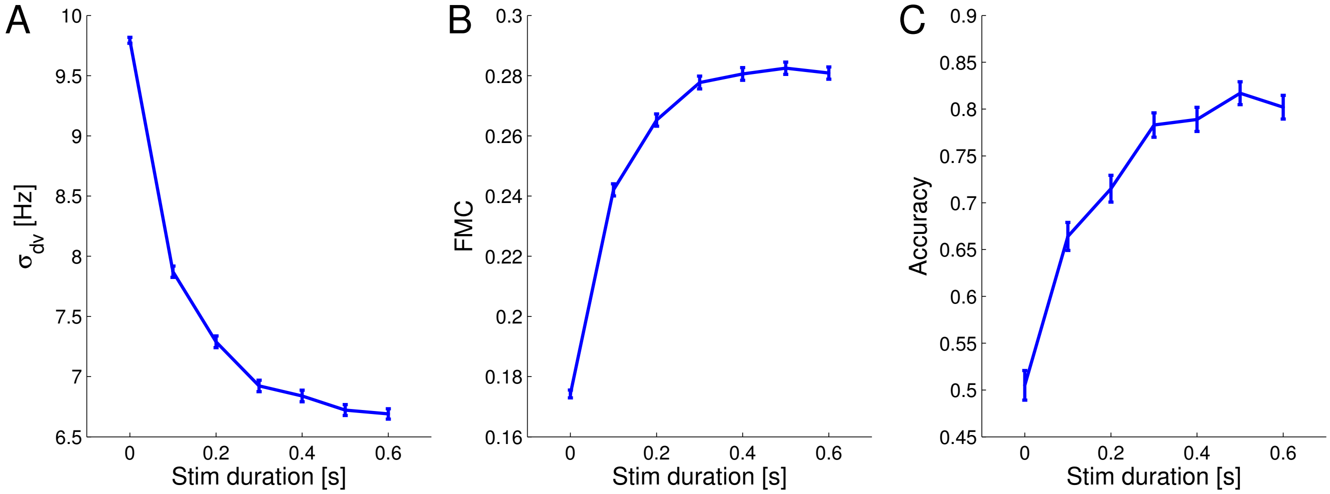


**Figure S9.** A $\sigma_{dv}$ as a function of the stimulus duration. B Selected option FMC as a function of the stimulus duration. C Performance as a function of stimulus duration.

## E Fixed delay task

The model was only tested on reaction time trials where it had to decide when it was ready and could sample the stimuli for as long as it needed. This was due to the fact that the behavioral data used to fit the psychophysical kernels was taken with reaction time trials. However, many experiments use the fixed delay paradigm where the stimulus is presented for a fixed time and the subject must respond [14,15]. Zylbergerg et al [16] also studied a task of this nature (random dot motion) and found that the decision and confidence kernels were qualitatively similar to those measured with the reaction time luminance discrimination task. However, they only tested one stimulus duration that was relatively long. When the stimuli are presented for very brief periods of time, subjective reports tend to be of low confidence. As the stimulus duration raises, so does the high confidence report rate [17–20]. Our model is also able to capture this fact (Fig. S9).

However, care must be taken when forcing our proposed network to decide upon a limited amount of evidence (short stimuli durations). If the sensory input disappears to soon, the network may have not yet committed to a choice. This implies that some of the modules may even revert to a low firing rate steady state and never select amongst the alternatives. We propose that once stimulation vanishes, if the network has not committed to a choice, it queries the modules (i.e. forces them to cast a vote). We propose this is accomplished by increasing the background task non-specific synaptic input ($I_{Background}$) to a level that forces the ANNs to choose one of the two alternatives.

We test our model's ability to explain confidence's dependence with stimulus duration by presenting it with two fluctuating sensory inputs. Both inputs are resampled from a normal distribution each 40ms. The target mean luminance is 52cd/m$^2$, the distractor mean is 50cd/m$^2$ and the standard deviation is 5cd/m$^2$. The stimuli are presented at $t = 1s$ and last different times. After the stimuli are turned off, the network is forced to select an option by increasing the background mean synaptic input to force a winner take all situation ($I_{Background} = 0.3455$nA). Upon the decision time, $\sigma_{dv}$ and FMC are measured (Fig. S9.A and B). The FMC increases ($\sigma_{dv}$ decreases) with stimulus duration and thus the probability of high confidence reports also increases. Accuracy also increases with stimulus duration until it reaches a stable value (Fig. S9.C).

This test is sufficient to say that when the environment controls stimulus duration, it modulates our model's confidence. However, if the model were aware of the length of the stimulation beforehand it could shift its criterium to favor speed or accuracy. This implies that the model could adopt a level of $I_{Background}$ that yielded slow or fast responses, thus tuning its speed/accuracy tradeoff [21,22]. We simulate 2000 trials with the same stimulation protocol as above but without turning off the sensory inputs, and for different $I_{Background}$ values. We observe, as is expected, that high $I_{Background}$ yields faster and less accurate responses, while low $I_{Background}$ produces slower, more accurate decision (Fig. S10.A and B). However, $\sigma_{dv}$ and FMC have a non-monotonic dependence with $I_{Background}$ (Fig. S10.C and D). Experimental observations show that subjects respond with greater confidence when favoring accurate responses [17,19]. To reproduce this, $\sigma_{dv}$ should monotonically increase with $I_{Background}$, which is clearly not the case. It is worthy to note that for $I_{Background}$ values above 0.3255nA (the value used for all simulations), $\sigma_{dv}$ (FMC) is monotonically increasing (decreasing), which could be a sign that our model can account for the speed accuracy trade-off only in a limited range of background inputs. Nevertheless, the non-monotonicity for $\sigma_{dv}$ as a function of $I_{Background}$ appears to be in grave contradiction with experimental observations; however, our model

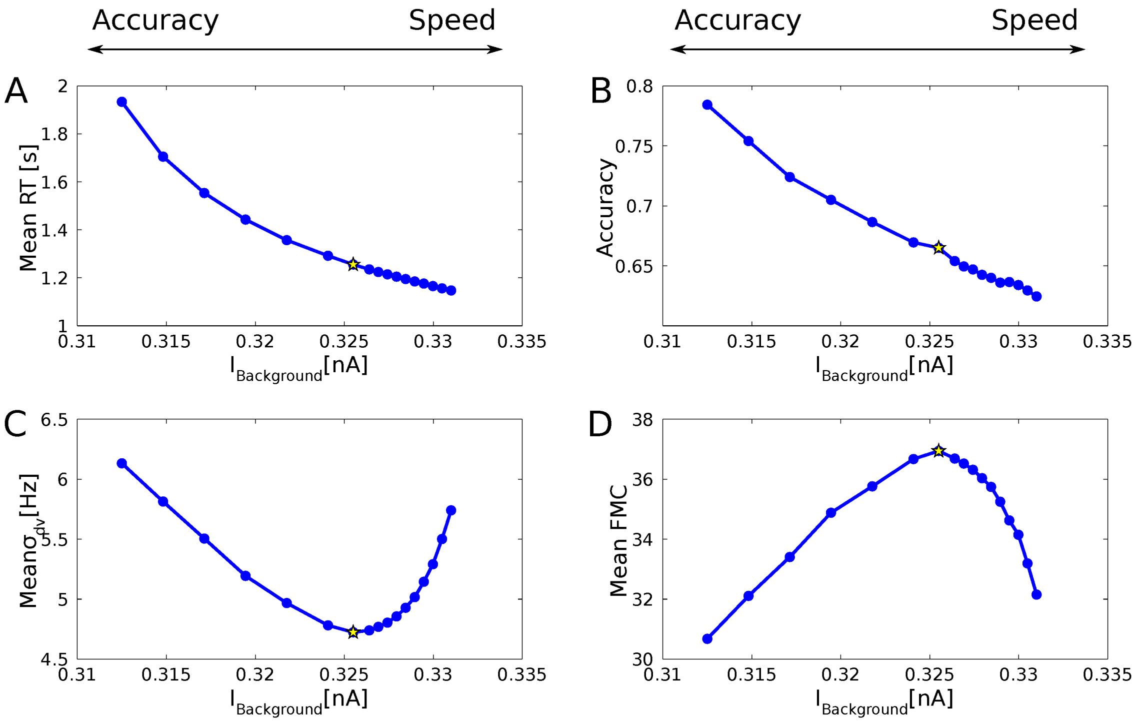


**Figure S10.** Simulations for different $I_{Background}$ values. A, B, C and D show the average RT, accuracy, $\sigma_{dv}$ and FMC, respectively, over 2000 independent trials. The star marker shows the data point that corresponds to $I_{Background} = 0.3255$nA, which is the background input used all other simulations.

is constructed assuming that the balance of speed and accuracy, i.e. the decision policy, is constant. Thus we do not study the problem of confidence calibration[23]. Calibration is the process through which a summary statistic (in our case the $\sigma_{dv}$ or FMC) is transformed to certainty level or a confidence report (which we assume occurs in a separate layer). This problem requires feedback connections and parameter tuning to actively learn the proper calibration for a variable decision policy. This also implies that the parameters that determine the probability of high confidence should also change with $I_{Background}$. This interesting problem is well beyond what we studied in the present work.

## References


1. Gardiner, C. W. *Handbook of Stochastic Methods: for Physics, Chemistry and the Natural Sciences* (Springer-Verlag Berlin Heidelberg New York, 1985).
2. Wang, X.-J. Probabilistic decision making by slow reverberation in cortical circuits. *Neuron* **36**, 955–68 (2002).
3. Wong, K.-F. & Wang, X.-J. A recurrent network mechanism of time integration in perceptual decisions. *The Journal of neuroscience : the official journal of the Society for Neuroscience* **26**, 1314–28 (2006).
4. Martí, D., Deco, G., Mattia, M., Gigante, G. & Del Giudice, P. A fluctuation-driven mechanism for slow decision processes in reverberant networks. *PloS one* **3**, e2534 (2008).
5. Uhlenbeck, G. E. & Ornstein, L. S. On the theory of the brownian motion. *Phys. Rev.* **36**, 823–841 (1930).
6. Gillespie, D. T. *Markov processes. An introduction for physical scientists* (Academic Press, INC., 1250 Sixth Avenue, San Diego, CA 92101, 1992).
7. Lo, C.-C. & Wang, X.-J. Cortico-basal ganglia circuit mechanism for a decision threshold in reaction time tasks. *Nature neuroscience* **9**, 956–63 (2006).
8. Carandini, M. & Heeger, D. J. Normalization as a canonical neural computation. *Nature reviews. Neuroscience* **13**, 51–62 (2012).
9. Chevalier, G. & Deniau, J. M. Disinhibition as a basic process of striatal functions. *Trends in Neurosciences* **13**, 277–280 (1990).
10. Rudolph, M., Pospischil, M., Timofeev, I. & Destexhe, A. Inhibition determines membrane potential dynamics and

controls action potential generation in awake and sleeping cat cortex. *The Journal of neuroscience : the official journal of the Society for Neuroscience* **27**, 5280–5290 (2007).

**11.** Azouz, R. & Gray, C. M. Stimulus-selective spiking is driven by the relative timing of synchronous excitation and disinhibition in cat striate neurons in vivo. *Eur J Neurosci* **28**, 1286–1300 (2008).

**12.** Cohen, M. X. & Frank, M. J. Neurocomputational models of basal ganglia function in learning, memory and choice. *Behavioural brain research* **199**, 141–56 (2009).

**13.** Letzkus, J. J. *et al.* A disinhibitory microcircuit for associative fear learning in the auditory cortex. *Nature* **480**, 331–335 (2011).

**14.** Mazurek, M. E., Roitman, J. D., Ditterich, J. & Shadlen, M. N. A role for neural integrators in perceptual decision making. *Cerebral Cortex* **13**, 1257–1269 (2003).

**15.** Kiani, R., Hanks, T. D. & Shadlen, M. N. Bounded integration in parietal cortex underlies decisions even when viewing duration is dictated by the environment. *The Journal of neuroscience : the official journal of the Society for Neuroscience* **28**, 3017–29 (2008).

**16.** Zylberberg, A., Barttfeld, P. & Sigman, M. The construction of confidence in a perceptual decision. *Frontiers in integrative neuroscience* **6**, 79 (2012).

**17.** Irwin, F. W., Smith, W. A. S. & Mayfield, J. F. Tests of two theories of decision in an ”expanded judgment” situation. *J Exp Psychol* **51**, 261–268 (1956).

**18.** Vickers, D. & Packer, J. Effects of alternating set for speed or accuracy on response time, accuracy and confidence in a unidimensional discrimination task. *Acta Psychologica* **50**, 179–197 (1982).

**19.** Vickers, D., Burt, J., Smith, P. & Brown, M. Experimental paradigms emphasising state or process limitations: I effects on speed-accuracy tradeoffs. *Acta Psychol* **59**, 129–161 (1985).

**20.** Vickers, D., Burt, J., Smith, P. & Brown, M. Experimental paradigms emphasising state or process limitations: Ii effects on confidence. *Acta Psychol* **59**, 163–193 (1985).

**21.** Bogacz, R., Wagenmakers, E.-J., Forstmann, B. U. & Nieuwenhuis, S. The neural basis of the speed-accuracy tradeoff. *Trends in neurosciences* **33**, 10–6 (2010).

**22.** Hanks, T. D., Kiani, R. & Shadlen, M. N. A neural mechanism of speed-accuracy tradeoff in macaque area lip. *eLife* **2014**, 1–17 (2014).

**23.** Meyniel, F., Sigman, M. & Mainen, Z. Confidence as bayesian probability: From neural origins to behavior. *Neuron* **88**, 78–92 (2015).
